# Properties of Hydrogen-Bonded Networks in Ethanol–Water Liquid Mixtures as a Function of Temperature: Diffraction Experiments and Computer Simulations

**DOI:** 10.1021/acs.jpcb.1c03122

**Published:** 2021-06-03

**Authors:** Szilvia Pothoczki, Ildikó Pethes, László Pusztai, László Temleitner, Koji Ohara, Imre Bakó

**Affiliations:** † Wigner Research Centre for Physics, Konkoly-Thege Miklós út 29-33, H-1121 Budapest, Hungary; ‡ International Research Organization for Advanced Science and Technology (IROAST), Kumamoto University, 2-39-1 Kurokami, Chuo-ku, Kumamoto 860-8555, Japan; § Research Centre for Natural Sciences, Magyar Tudósok Körútja 2, H-1117 Budapest, Hungary; ∥ Diffraction and Scattering Division, JASRI, SPring-8, 1-1-1, Kouto, Sayo-cho, Sayo-gun, Hyogo 679-5198, Japan

## Abstract

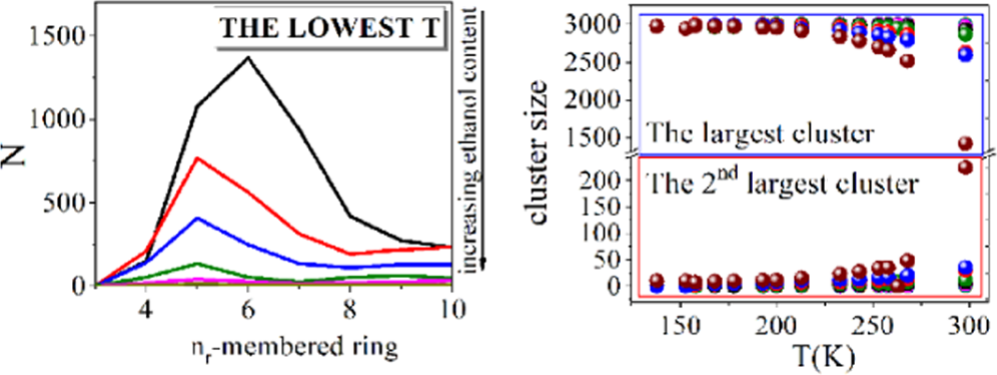

New X-ray and neutron diffraction experiments have been performed on ethanol–water mixtures as a function of decreasing temperature, so that such diffraction data are now available over the entire composition range. Extensive molecular dynamics simulations show that the all-atom interatomic potentials applied are adequate for gaining insight into the hydrogen-bonded network structure, as well as into its changes on cooling. Various tools have been exploited for revealing details concerning hydrogen bonding, as a function of decreasing temperature and ethanol concentration, like determining the H-bond acceptor and donor sites, calculating the cluster-size distributions and cluster topologies, and computing the Laplace spectra and fractal dimensions of the networks. It is found that 5-membered hydrogen-bonded cycles are dominant up to an ethanol mole fraction *x*_eth_ = 0.7 at room temperature, above which the concentrated ring structures nearly disappear. Percolation has been given special attention, so that it could be shown that at low temperatures, close to the freezing point, even the mixture with 90% ethanol (*x*_eth_ = 0.9) possesses a three-dimensional (3D) percolating network. Moreover, the water subnetwork also percolates even at room temperature, with a percolation transition occurring around *x*_eth_ = 0.5.

## Introduction

The physicochemical properties of water–ethanol solutions have been among the most extensively studied subjects in the field of molecular liquids over the past few decades,^1−17^ due to their high biological and chemical significance. Even though they are composed of two simple molecules, the behavior of their hydrogen-bonded network structures can be very complex, due to the competition between hydrophobic and hydrophilic interactions.^18−25^ The characteristics of these networks can be greatly influenced by the concentration. Usually, three regions of the composition range are distinguished qualitatively: the water-rich, the medium or transition, and the alcohol-rich regions.

Most thermodynamic properties, such as excess enthalpy, isentropic compressibility, and entropy, show either maxima or minima in the low-alcohol-concentration region (molar ratio of ethanol *x*_eth_< 0.2).^26−28^ Differential scanning calorimetry, nuclear magnetic resonance (NMR), and infrared (IR) spectroscopic studies suggested a transition point around *x*_eth_ = 0.12, while additional transition points were found at *x*_eth_ = 0.65 and 0.85.^29−31^ Concerning the intermediate region around *x*_eth_ = 0.5, a maximum was observed by the Kirkwood–Buff integral theory, which suggests water–water aggregation.^32−35^ Also, a maximum of the concentration fluctuations was found in the same region, at *x*_eth_ = 0.4, by small-angle X-ray scattering.^36^

Quite recently, we studied structural changes in ethanol–water mixtures as a function of temperature in the water-rich region (up to *x*_eth_ = 0.3).^23,24^ There we focused mainly on the cyclic entities. We found that the number of hydrogen-bonded rings increased upon lowering the temperature, and that fivefold rings were in majority, especially at *x*_eth_ > 0.1 ethanol concentrations.

In the present study, we extend both X-ray diffraction (XRD) measurements and molecular dynamics simulations to investigate ethanol–water mixtures down to their freezing points, over the entire ethanol concentration range. Furthermore, new neutron diffraction experiments have been performed in the water-rich region (up to *x*_eth_ = 0.3). These neutron data fit nicely in the present line of investigation and support our earlier findings. The main goal here was to provide a complete picture of the behavior of the hydrogen-bonded network over the entire composition range in ethanol–water mixtures, between room temperature and the freezing point. In order to identify the existence and the location of the percolation threshold, we monitor the changes of the number of molecules acting as donor or acceptor, cluster-size distributions, cyclic and noncyclic properties, and the Laplace spectra of the H-bonded network.

## Methods

### X-Ray and Neutron Diffraction Experiments

Series of samples of ethanol–water mixtures have been prepared with natural isotopic abundances for synchrotron X-ray (ethanol: Sigma-Aldrich, better than 99.9% purity), and with fully deuterated forms of both compounds for neutron diffraction experiments (Sigma-Aldrich; C_2_D_5_OD: deuterium content higher than 99.5%; D_2_O: deuterium content higher than 99.9%). In the absence of suitable experimental data, low-temperature densities have been determined by molecular dynamics simulations (see below) in the NPT ensemble. Numerical data are reported in Table S2; as is clear from the table, this method has proven to be accurate within 1% at room temperature.

Synchrotron experiments were performed at the BL04B2^37^ high-energy X-ray diffraction beamline of the Japan Synchrotron Radiation Research Institute (SPring-8, Hyogo, Japan). Diffraction patterns could be obtained over a scattering variable, *Q*, ranging between 0.16 and 16 Å^–1^, for samples with alcohol contents of 40, 50, 60, 70, 80, 85, 90, and 100 mol % of ethanol (*x*_eth_ = 0.4, 0.5, 0.6, 0.7, 0.8, 0.85, 0.9, and 1.0, respectively). Diffraction patterns have been recorded starting from room temperature and cooling down to the freezing point for each composition.

Neutron diffraction measurements have been carried out at the 7C2 diffractometer of Laboratoire Léon-Brillouin.^38^ Details of the experimental setup, the applied ancillary equipment, and data correction procedure were already reported^25^ for methanol–water samples measured under the same conditions. For both neutron and X-ray raw experimental data, standard procedures^38,39^ have been applied during data treatment.

**Figure 1:**
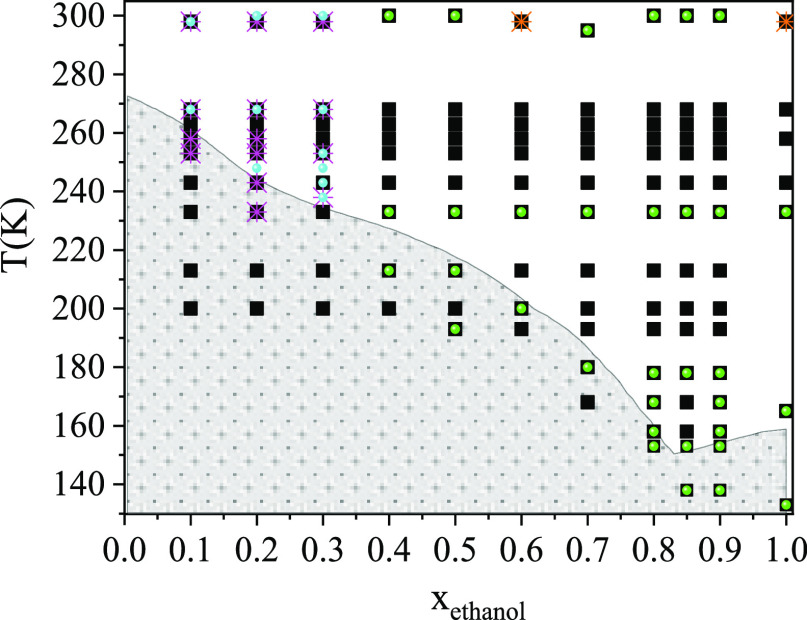
Phase diagram of ethanol–water mixtures. Gray area: solid state; white area: liquid state (as these values were obtained from ref (40)). Black solid squares: present MD simulations; green solid circles: new X-ray diffraction data sets; light-blue solid circles: new neutron diffraction data sets; orange crosses: X-ray diffraction data sets from ref (19); magenta crosses: X-ray diffraction data sets from ref (21).

**Figure 2:**
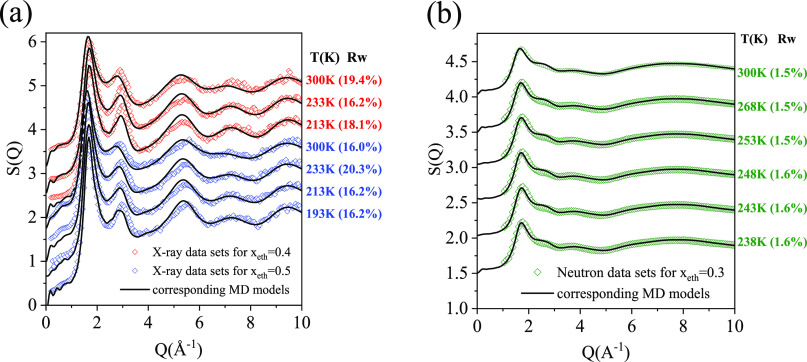
Measured and calculated TSSFs (a) for X-ray diffraction, and (b) for neutron diffraction.

All temperature and composition points visited by the new X-ray and neutron diffraction experiments are displayed together with the phase diagram of ethanol–water mixtures in Figure 1. Total scattering structure factors (TSSFs) obtained from the new experimental data are shown in Figures 2 and S1.

### Molecular Dynamics (MD) Simulations

Molecular dynamics (MD) simulations were carried out using the GROMACS software^41^ (version 2018.2). The Newtonian equations of motions were integrated by the leapfrog algorithm, using a time step of 2 fs. The particle-mesh Ewald algorithm was used for handling long-range electrostatic forces.^42,43^ The cutoff radius for nonbonded interactions was set to 1.1 nm (11 Å). For ethanol molecules, the all-atom optimized potentials for liquid simulations (OPLS-AA)^44^ force field was used. Bond lengths were kept fixed using the LINCS algorithm.^45^ The parameters of atom types and atomic charges can be found in Table S1. Based on the results of our earlier study,^22^ the TIP4P/2005^46^ water model was applied, as handled by the SETTLE algorithm.^47^ For each composition, 3000 molecules (with respect to compositions and densities) were placed in a cubic box, with periodic boundary conditions. The box lengths, together with corresponding bulk densities, can be found in Table S2. All MD models studied are shown in Figure 1. Table S3 shows the various phases of the MD simulations.

## Results and Discussion

### Total Scattering Structure Factors

Total scattering structure factors have been calculated from MD trajectories via the standard route, see e.g., ref (22) (also, see the Supporting Information). As typical examples, total scattering structure factors obtained from measured X-ray diffraction signals, for mixtures with *x*_eth_ = 0.4 and 0.5, as a function of temperature are shown in Figure 2a. Similarly, Figure 2b shows TSSFs from neutron diffraction for *x*_eth_ = 0.3. Calculated TSSFs are also presented in Figure 2. Additional measured TSSFs, together with the corresponding calculated TSSFs, can be found in Figure S1.

The agreement between calculated and measured TSSFs for neutron diffraction appears to be almost perfect. In the case of X-ray diffraction, apparent differences can be observed, mostly around the second maximum. *R*_w_ factors were calculated to characterize the differences between MD simulated [*F*^S^(*Q*)] (averaged over many time frames) and experimental structure factors [*F*^E^(*Q*)] quantitatively, thus providing a kind of goodness-of-fit (c.f. Supporting Information). Note that values of *R*_w_ for the two different experimental methods are not compared, due to the different data treatment procedures. It can be stated that MD models are appropriate for further analyses.

We note here that detailed analyses of partial radial distribution functions (PRDFs) are not within the scope of the present work; however, all PRDFs related to H-bonding properties are shown in Figures S2–S8.

### H-Bond Acceptors and Donors

The calculated average hydrogen-bond numbers for the entire mixture and for the ethanol subsystem can be found in Figures S9–S11. The H-bond definition applied is presented also in the Supporting Information. All of the following analyses (together with the identification of cyclic and noncyclic entities) were performed using our in-house computer code.^48^

The molecules participating in H-bonds can be classified into two groups according to their roles as proton acceptors or donors. Each molecule may have a certain number of donor sites (*n*_D_) and a certain number of acceptor sites (*n*_A_), and thus can be characterized by the “*n*_D_D:*n*_A_A” combination. For example, 1D:2A denotes a molecule that acts as a donor of 1 H-bond and accepts 2 H-bonds. The sum of *n*_D_ and *n*_A_ for a given molecule provides the number of H-bonds (*n*_HB_) of that molecule (c.f. Figure S9).

**Figure 3:**
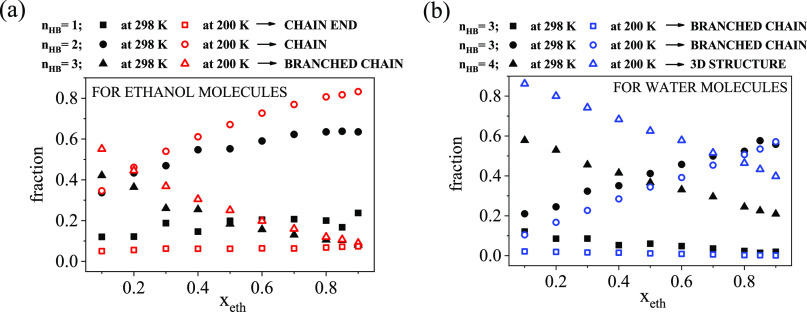
Donor and acceptor sites (a) for ethanol molecules and (b) for water molecules as a function of ethanol concentration.

The most populated fractions for ethanol molecules are 1D:1A, 1D:2A and the sum of 0D:1A and 1D:0A (Figure 3a), while for water molecules, they are 1D:2A, 2D:1A, and 2D:2A (Figure 3b). These groups altogether contain 80% of all H-bonds at room temperature and 90% of all H-bonds at low temperatures.

Concerning ethanol molecules, the occurrence of the 1D:1A combination is above 50% over almost the entire concentration range, independently of the temperature. This group corresponds to chain-like arrangements, which become more preferred with increasing ethanol content and at lower temperatures. It is remarkable that in the water-rich region, the 1D:2A combination has the same (*x*_eth_ = 0.2) or even a slightly higher (*x*_eth_ = 0.1) occurrence than that of 1D:1A. With decreasing water content, the occurrence of 1D:2A decreases. Also, this group is more dominant at 200 K.

Water molecules most often behave according to the 2D:2A scheme. The occurrence of this arrangement significantly increases with decreasing temperature, as well as with increasing water content. On the other hand, the fractions of 1D:2A and 2D:1A combinations increase as the temperature increases. There is a well-defined asymmetry between these two (1D:2A and 2D:1A) types of water molecules in terms of their populations, and the difference becomes more pronounced with increasing ethanol concentration. The fractions of 1A:1D for ethanol molecules, 1D:2A for ethanol molecules, 2D:1A for water molecules, and 2D:2A for water molecules as a function of temperature can be found in Figure S12. Furthermore, the calculated H-bond number excess parameter is shown in Figure S13. A well-defined maximum can be identified for *f*_wat–wat_ around ethanol mole fractions of 0.5–0.6 at 298 K, which is shifted at 200 K to ethanol mole fractions of 0.6–0.7. This may be attributed to a significant number of excess water molecules in the solvation shell of water. This maximum agrees well with the maximum of *G*_wat–wat_ in Kirkwood–Buff integral theory.^32−35^

### Clustering and Percolation

Two molecules are regarded as members of a cluster, according to the definitions introduced by Geiger et al.,^49^ if they are connected by a chain of hydrogen bonds. Concerning the pure components of the mixtures studied here, water molecules form a three-dimensional (3D) percolating hydrogen bonding network,^46,49^ whereas in pure ethanol, only chain (or branched chain) structures can be detected.^50,51^

There are several descriptors connected to the properties of networks that can be used for the determination of the percolation transition. This work focuses on the cluster-size distribution (*P*(*n*_c_)). However, very similar conclusions may be drawn from scrutinizing several other parameters such as the average largest cluster size (C1), average second largest cluster size (C2), and the fractal dimension of the largest cluster (*f*_d_). A more detailed discussion is provided in the Supporting Information, Figure S14.

**Figure 4:**
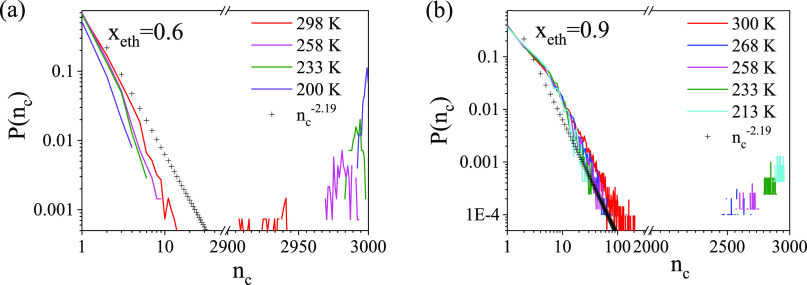
Cluster-size distributions from the room temperature to the lowest studied temperature (a) for *x*_eth_ = 0.6 and (b) for *x*_eth_ = 0.9.

Cluster-size distributions are shown in Figure 4. The system is percolated when the number of molecules in the largest cluster is in the order of the system size. For random percolation on a 3D cubic lattice, the cluster-size distribution can be given by *P*(*n*_c_) = *n*_c_^–2.19^, where *n*_c_ is the number of molecules in a given cluster.^52,53^ Percolation transition can be ascertained by comparing the calculated cluster-size distribution function of the present system with that obtained for the random one. At each temperature, up to *x*_eth_ = 0.85 (Figures 4 and S15a) a well-defined contribution can be found at large cluster-size values, signaling percolation. Systems with *x*_eth_ = 0.9 (Figure 4b) show the same behavior at lower temperatures, but this signature disappears at room temperature. This suggests that in the latter case, the system is close to the percolation threshold, which can be expected between 0.9 and 1.0 ethanol molar fraction. Ethanol molecules in the pure liquid compose nonpercolated assemblies (Figure S15b).^2,8,20^ The structural disintegration of the percolated H-bonded network can be connected to the transition point found at *x*_eth_ = 0.85 by NMR and IR spectroscopic studies.^29−31^

**Figure 5:**
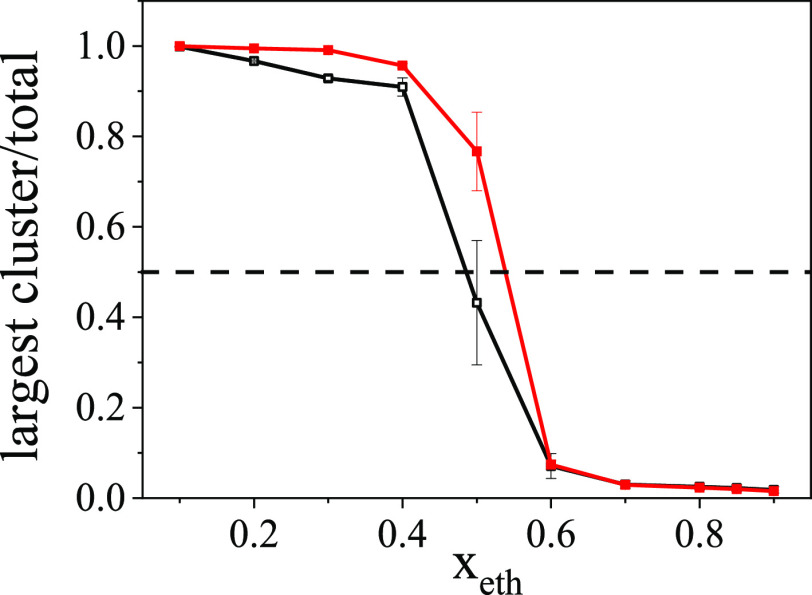
Average largest cluster size divided by the total number of water molecules, as a function of the ethanol concentration. Black open square symbols: 300 K; red solid square symbols: 200 K.

The role of water molecules was then analyzed separately. All of the four quantities mentioned above for characterizing the percolation transition were calculated, taking into account only the H-bonds between water molecules. Figure 5 shows one representation. The average largest cluster size divided by the total number of water molecules drops to below 0.5, which indicates the percolation transition between *x*_eth_ = 0.4 and 0.5 at 300 K, and between *x*_eth_ = 0.5 and 0.6 at 200 K, respectively. This behavior can be compared to the maximum value observed by the Kirkwood–Buff integral theory.^32−35^ Similar values were found for percolation in formamide–water^54^ and glycerol–water mixtures.^55^ However, in those cases, both of the constituents (not only water molecules) form 3D percolating H-bonded networks in the liquid state. Here, in contrast, independently of the concentration, ethanol molecules form only short chain-like structures, but not large percolated networks. Typical hydrogen-bond network topologies for the largest cluster at compositions of *x*_eth_ = 0.4, 0.7, and 0.9 are shown in Figure S16.

### Rings and Chains

Hydrogen-bonded clusters may contain noncyclic (or “chain-like,” either linear or branched) and cyclic (“closed into themselves”) entities (c.f. Figure S16). The number of cyclic entities (*N*_cycl_), the number of molecules (*N*_noncycl_) that are not members of any ring (*n*_c_ < 10), and the cyclic size distribution (*n*_r_) were calculated using the algorithms developed by Chihaia et al.^56^

**Figure 6:**
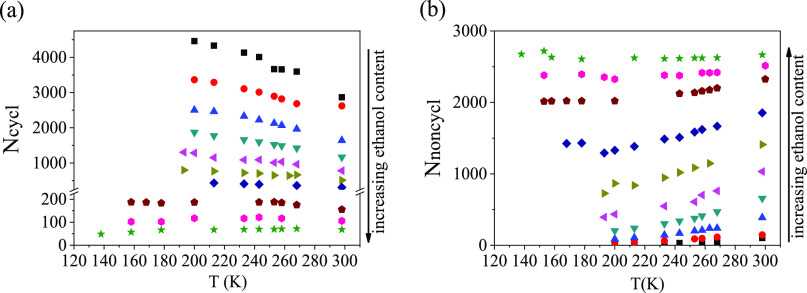
(a) Number of cyclic entities and (b) number of noncyclic entities as a function of ethanol concentration and temperature. Box solid: *x*_eth_ = 0.1; red circle solid: *x*_eth_ = 0.2; blue triangle up solid: *x*_eth_ = 0.3; dark cyan triangle down solid: *x*_eth_ = 0.4; magenta triangle left-pointing solid: *x*_eth_ = 0.5; dark yellow triangle right-pointing solid: *x*_eth_ = 0.6; navy blue diamond solid: *x*_eth_ = 0.7; dark red pentagon solid: *x*_eth_ = 0.8; dark magenta hexagon solid: *x*_eth_ = 0.85; green star solid: *x*_eth_ = 0.9. (Note that in most cases, there is more than just one cycle that crosses a given molecule. This is a particularly prominent feature in the case of water molecules that can easily span a genuine 3D network. This is how the number of rings can exceed the number of molecules).

Figure 6 summarizes the numbers of cyclic and noncyclic entities as a function of ethanol concentration and temperature. The number of cycles decreases significantly with increasing ethanol content. As a result, in the ethanol-rich region (above 70 mol %), mostly noncyclic entities are present. Both *N*_noncycl_ and *N*_cycl_ show a strong temperature dependence up to around *x*_eth_ = 0.80–0.85. This effect appears to be more pronounced for noncyclic entities. This composition may be, again, linked to a transition point detected by differential scanning calorimetry, NMR, and IR spectroscopic studies.^29−31^ At the highest ethanol concentrations, where most of the molecules are arranged in chains, the number of chains formed is independent of temperature.

**Figure 7:**
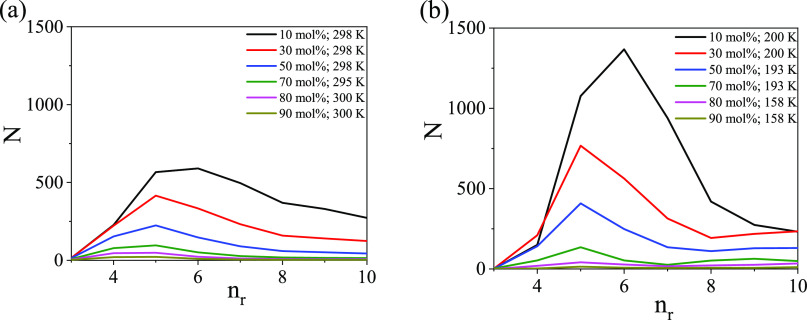
Ring-size distributions as a function of *x*_eth_. (a) At room temperature and (b) at the studied lowest temperature.

It has already been demonstrated that in pure water, molecules prefer to form six-membered rings at room temperature, and that this behavior becomes more pronounced during cooling.^24^ This statement remains true in ethanol–water mixtures (Figure 7) as well, as long as the ethanol molar ratio stays around 0.1, whereas for *x*_eth_ = 0.2 and 0.3, 5-membered rings become dominant.^22^ The composition where this significant change of the preferred ring size occurs corresponds well with another anomalous transition point found in this liquid mixture,^19−21^ and perhaps even more significantly, with the extrema found in terms of excess enthalpy, isentropic compressibility, and entropy.^26−28^ Regardless of the ethanol concentration, there are always more rings at low temperatures.^22,24^

Focusing now on mixtures with ethanol contents higher than 30 mol % (*x*_eth_ = 0.3), 5-membered rings take the leading role up to a concentration somewhere between *x*_eth_ = 0.7 and 0.8, where the number of rings (per particle configuration) falls below 100. These tendencies are more pronounced at lower temperatures.

Note that for the sake of comparison, results for the region between *x*_eth_ = 0.1 and 0.3 are also presented in Figure 7, although a detailed discussion of the ring-size distributions for the water-rich region can be found in ref (22). The new feature here is that the corresponding curves are consistent also with our fresh neutron diffraction data (cf. Figure 2b).

### Spectral Properties of H-Bonded Networks

It has already been shown that the Laplace spectra^57−67^ of H-bonded networks are a good topological indicator for monitoring the percolation transition in liquids.^68^ Several authors have studied the relationship between the eigenvector corresponding to the second smallest eigenvalue (λ_2_) and the graph structure; well-documented reviews can be found in the literature.^58,62,65^ More details are available in the Supporting Information.

**Figure 8:**
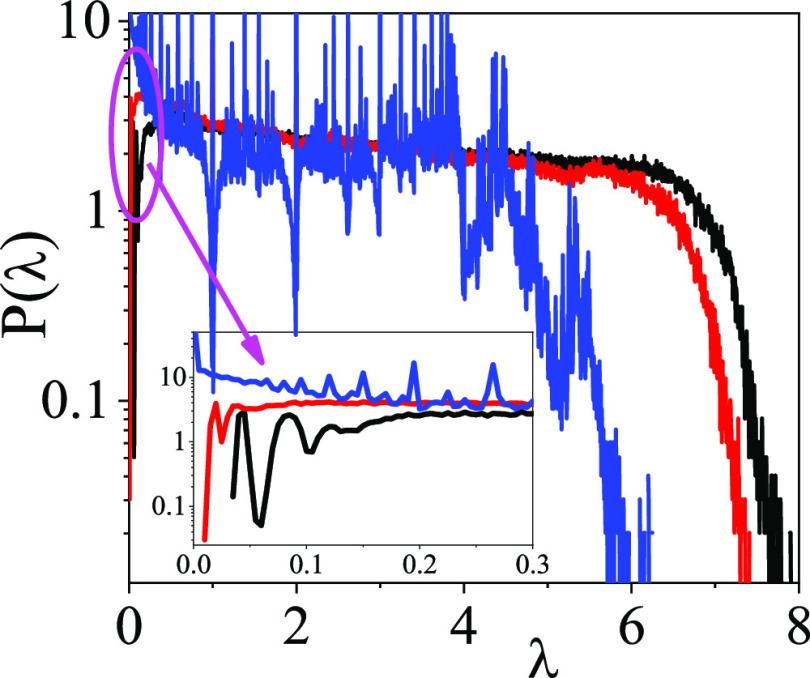
Laplace spectra of ethanol–water mixtures as a function of concentration at room temperature. Black line: *x*_eth_ = 0.1; red line: *x*_eth_ = 0.4; and blue line: *x*_eth_ = 0.95.

Figure 8 provides the Laplace spectra of ethanol–water mixtures as a function of concentration at room temperature. The low λ values (up to 0.3) are enlarged at the bottom. Spectra of the pure constituents can be found in ref (68). According to the topology of the H-bonded network, two cases can be distinguished in connection with the Laplace spectra: (1) For liquids whose molecules form a 3D percolated network, a well-defined gap can be detected at low eigenvalues. (2) For systems without an extended 3D network structure, where molecules link to each other so as to construct (branched) chains, several well-defined peaks (λ = 0.5, 1, 1.5, 2,...) show up, without any recognizable gap at low eigenvalues. Pure water falls into the first category, while pure liquid ethanol belongs to the second one.^68^

Concerning the mixtures studied here, the existence of the gap mentioned above depends on composition. Up to an ethanol content of 95%, a well-defined gap can be found. However, above 95% ethanol content, this gap disappears. Regarding the H-bond network, this means that there is a limiting alcohol concentration beyond which the presence of chains of molecules is dominant. In this concentration region, percolated networks cannot be detected. That given concentration at which the percolation vanishes can be considered as a percolation threshold. At concentrations lower than this limiting value, water-like 3D percolated networks are formed. At lower temperatures, no percolation threshold could be found and all systems have 3D network structures (cf. Figure 4b).

## Summary and Conclusions

X-ray and neutron diffraction measurements have been conducted on ethanol–water mixtures, as a function of temperature, down to the freezing points of the liquids. As a result of the new experiments, temperature-dependent X-ray structure factors are now available for the entire composition range.

For interpreting the experimental data, series of molecular dynamics simulations have been performed for ethanol–water mixtures with ethanol contents between 10 and 90 mol % (between *x*_eth_ = 0.1 and 0.9). The temperature was varied between room temperature and the freezing point of the actual mixture. With the aim of evaluating the applied force fields, MD models were compared to new X-ray diffraction data over the entire composition range, as well as to new neutron diffraction experiments over the water-rich region. It has been established that the combination of OPLS-AA (ethanol) and TIP4P/2005 (water) potentials has reproduced individual experimental data sets, as well as their temperature dependence, with a more-than-satisfactory accuracy. It may therefore be justified that the MD models are used for characterizing hydrogen-bonded networks that form in ethanol–water mixtures.

When the H-bond acceptor and donor roles of water molecules are taken into account, the occurrence of the 2D:2A combination increases linearly at every concentration with decreasing temperature.

The percolation threshold and its variation with temperature have been estimated via various approaches: we found that even at the highest alcohol concentration, the entire system percolates at low temperatures. The percolation transition for the water subsystems was found to be a 3D percolation transition that occurs between *x*_eth_ = 0.4 and 0.5 at 300 K, and between *x*_eth_ = 0.5 and 0.6 at 200 K, respectively. These values resonate well with the extrema found by the Kirkwood–Buff theory,^32−35^ and by small-angle scattering experiments.^36^

Concerning the topology of H-bonded assemblies, in mixtures with ethanol contents higher than 30 mol % (*x*_eth_ = 0.3), a 5-membered ring takes the leading role up to *x*_eth_ = 0.7 and 0.8, where the number of rings falls dramatically. This tendency is most pronounced at low temperatures. The composition (about *x*_eth_ = 0.2) where 5-membered rings become dominant matches perfectly the composition where the extrema of many thermodynamic quantities have been identifed.^26−28^

## Supporting Information Available

Measured and calculated total scattering structure factors for X-ray diffraction for the ethanol–water mixtures as a function of temperature (Figure S1); Lennard–Jones parameters and partial charges for the atom types of ethanol used in the MD simulations (Table S1); steps of molecular dynamics simulation at each studied temperature (Table S2); box lengths (nm), corresponding bulk densities (g/cm^3^) for each simulated system (Table S3); selected partial radial distribution functions for the mixture with 40, 50, 60, 70, 80, 85 and 90 mol % ethanol as a function of temperature (Figures S2–S8); average H-bond numbers considering each molecule, regardless of their types, together with the case when considering water–water H-bonds only (Figure S9); average H-bond number for ethanol–ethanol subsystem (Figure S10); average H-bond numbers considering connections of water molecules only, as well as considering connections of ethanol molecules only (Figure S11); fraction of donor and acceptor sites as a function of temperature (Figure S12); H-bond number excess parameter (Figure S13); the average largest cluster size (C1) and average second largest cluster size (C2) as a function of ethanol concentration and temperatures in ethanol–water mixtures (Figure S14); cluster-size distributions from the room temperature to the lowest studied temperature (a) for *x*_eth_ = 0.85, (b) for pure ethanol (Figure S15); typical hydrogen-bonded network topologies in water–ethanol mixtures at concentrations *x*_eth_ = 0.40 (left), 0.70 (middle), and 0.90 (right) (Figure S16); values of the inequality calculated by eq 4; black open squares: left side of eq 4 at 298 K; black solid squares: right side of eq 4 at 298 K; red open circles: left side of eq 4 at 233 K; red solid circles: right side of eq 4 at 233 K (Figure S17) (PDF)
