# Supplementary material for: Properties of Hydrogen-Bonded Networks in Ethanol–Water Liquid Mixtures as a Function of Temperature: Diffraction Experiments and Computer Simulations
Source: J Phys Chem B. 2021 Jun 3;125(23):6272–9. doi: 10.1021/acs.jpcb.1c03122 (PMC8279560; doi:10.1021/acs.jpcb.1c03122)
Supplement: Supplementary file 1 — jp1c03122_si_001.pdf [file jp1c03122_si_001.pdf]

Supporting Information

for

Properties of Hydrogen Bonded Networks in Ethanol-water Liquid Mixtures as a Function of Temperature: Diffraction Experiments and Computer Simulations

Szilvia Pothoczki[1], Ildikó Pethes[1], László Pusztai[1,2], László Temleitner[1], Koji Ohara[4], and Imre Bakó*[3]*

[1]Wigner Research Centre for Physics, H-1121 Budapest, Konkoly Thege M. út 29-33., Hungary

[2]International Research Organization for Advanced Science and Technology (IROAST), Kumamoto University, 2-39-1 Kurokami, Chuo-ku, Kumamoto, 860-8555, Japan

[3]Research Centre for Natural Sciences, H-1117 Budapest, Magyar tudósok körútja 2., Hungary

[4]Diffraction and Scattering Division, JASRI, Spring-8, 1-1-1, Kouto, Sayo-cho, Sayo-gun, Hyogo 679-5198, Japan

*E-mail: pothoczki.szilvia@wigner.hu; Phone: +36 1 392 2222/ext. 1469

*E-mail: bako.imre@ttk.hu; Phone: +36 1 382 6981

Calculation of the total scattering structure factor from molecular dynamics trajectories

The part of the total structure function that provides a description for the liquid structure may be calculated from the partial rdf's according to the equation

$$F(Q) = \sum_{\alpha \geq \beta}\sum \frac{(2 - \delta_{\alpha\beta}) x_\alpha x_\beta f_\alpha f_\beta h_{\alpha\beta}(Q)}{M(Q)}$$

Here, f_α is the scattering length (in neutron diffraction) or scattering factor (X-ray diffraction) of atom type α; f_α depends on Q in the case of X-ray diffraction, and is constant in the case of neutron diffraction. x_α is the corresponding mole fraction. $M(Q) = (\sum_{\alpha=1}^{n} x_\alpha f_\alpha)^2$, $h_{\alpha\beta}(Q)$, the partial structure factor, is defined from the partial rdf's, $g_{\alpha\beta}(r)$, according to the following equation:

$$h_{\alpha\beta}(Q) = 4\pi\rho \int_0^{r_{max}} r^2 (g_{\alpha\beta}(r) - 1) \frac{\sin(Qr)}{Qr} dr$$

where ρ is the atomic number density of the liquid.

Figure S1: Measured and calculated total scattering structure factors (TSSF's) for the ethanol-water mixtures as a function of temperature. a) X-ray diffraction TSSF's for x_{eth}=0.8 and 0.85; b) X-ray diffraction TSSF's for x_{eth}=0.9 and 1.0; c) X-ray diffraction TSSF's for x_{eth}=0.6 and 0.7; d) neutron diffraction TSSF's for x_{eth}=0.1 and 0.2.

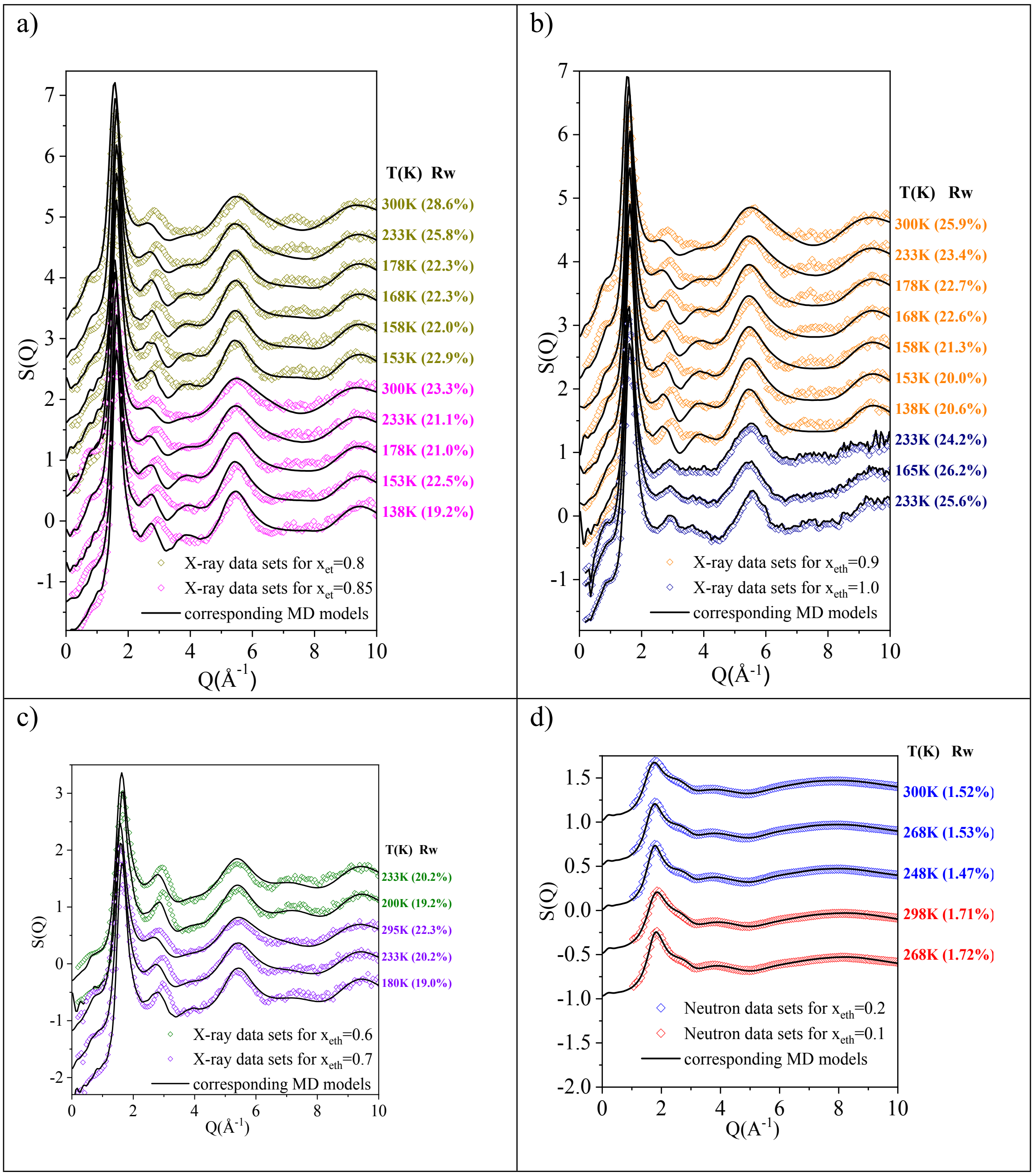

Table S1: Lennard-Jones parameters and partial charges for the atom types of ethanol used in the MD simulations.

Atom types	σ (Å)	ε (kJ/mol)	q (e)
C (H_3)	3.5	0.276144	-0.18
C (H_2)	3.5	0.276144	0.145
H	2.5	0.12552	0.06
O	3.12	0.71128	-0.683
OH	0	0	0.418

Table S2: The first rows: box lengths (nm), the second rows: the corresponding bulk densities (g/cm^3) for each simulated system. One star symbol: at 298; two stars symbol: at 295 K; without symbol: at 300 K. The third rows contain the experimental densities (g/cm^3) at 298.15 K.[S1]

x_{eth}	0.1	0.2	0.3	0.4	0.5	0.6	0.7	0.8	0.85	0.9	1.0
298 K	4.750*	4.998*	5.243*	5.479	5.685	5.865*	6.052**	6.247	6.354	6.441	6.608*
	0.9678	0.9429	0.9138	0.8855	0.8687	0.8606	0.8462	0.8268	0.8129	0.8068	0.7953
	0.9632	0.9337	0.9047	0.8799		0.8409	0.8247	0.8105		0.7978	0.7854
268 K	4.741	4.961	5.186	5.412	5.614	5.810	6.007	6.175	6.275	6.348	6.517
	0.9731	0.9638	0.9440	0.9187	0.9023	0.8850	0.8656	0.8559	0.8439	0.8425	0.8293
263 K	4.730	4.959	5.179	5.383	5.617	5.801	5.984	6.166	6.271	6.332	X
	0.9799	0.9652	0.9479	0.9339	0.9009	0.8895	0.8754	0.8596	0.8456	0.8491	
258 K	4.726	4.951	5.183	5.393	5.603	5.792	5.976	6.146	6.244	6.331	6.498
	0.9826	0.9699	0.9456	0.9287	0.9077	0.8935	0.8789	0.8682	0.8565	0.8495	0.8363
253 K	4.718	4.946	5.175	5.381	5.577	5.765	5.973	6.124	6.224	6.313	X
	0.9878	0.9729	0.9501	0.9349	0.9201	0.9059	0.8804	0.8775	0.8651	0.8568	
243 K	4.723	4.924	5.151	5.371	5.558	5.759	5.948	6.117	6.212	6.297	6.444
	0.9848	0.9857	0.9635	0.9403	0.9299	0.9090	0.8912	0.8805	0.8698	0.8631	0.8578
233 K	4.713	4.928	5.137	5.355	5.545	5.742	5.933	6.095	6.189	6.259	6.429

(S1) Gonzalez, B.; Calvar, N.; Gomez, E.; Dominguez A. Density, dynamic viscosity, and derived properties of binary mixtures of methanol or ethanol with water, ethyl acetate, and methyl acetate at T = (293.15, 298.15, and 303.15) K. *J. Chem. Thermodynamics* **2007,** 39, 1578.

	0.9905	0.9835	0.9715	0.9487	0.9363	0.9171	0.8983	0.8901	0.8799	0.8788	0.8636
213 K	4.704<br>0.9964	4.905<br>0.9975	5.115<br>0.9840	5.312<br>0.9717	5.511<br>0.9536	5.688<br>0.9432	5.886<br>0.9201	6.049<br>0.9105	6.132<br>0.9046	6.220<br>0.8956	X
200 K	4.702<br>0.9976	4.892<br>1.0053	5.096<br>0.9948	5.300<br>0.9782	5.485<br>0.9672	5.674<br>0.9502	5.851<br>0.9365	6.015<br>0.9260	6.112<br>0.9132	6.188<br>0.9096	X
193 K	X	X	X	X	5.473<br>0.9739	5.670<br>0.9524	5.841<br>0.9415	6.010<br>0.9285	6.092<br>0.9223	6.172<br>0.9169	X
180 K	X	X	X	X	X	X	5.826<br>0.9488	X	X	X	X
178 K	X	X	X	X	X	X	X	5.974<br>0.9455	6.073<br>0.9309	6.151<br>0.9261	X
168 K	X	X	X	X	X	X	5.819<br>0.9519	5.972<br>0.9463	6.067<br>0.9338	6.147<br>0.9278	X
165 K	X	X	X	X	X	X	X	X	X	X	6.295<br>0.9199
158 K	X	X	X	X	X	X	X	5.970<br>0.9471	6.051<br>0.9413	6.121<br>0.9400	X
153 K	X	X	X	X	X	X	X	X	6.043<br>0.9451	6.098<br>0.9505	X
138 K	X	X	X	X	X	X	X	X	6.029<br>0.9517	6.082<br>0.9581	X
133 K	X	X	X	X	X	X	X	X	X	X	6.259<br>0.9358

Table S3: Steps of Molecular Dynamics simulation at each studied temperature. (After energy minimization the initial box was heated up to 340 K in order to avoid the aggregation of ethanol molecules. The stages of the simulation series for each composition were the following at each studied temperature (even at 340 K).)

Type	Run time (ns)	Thermostat	time const. τ_T (ps)	Barostat	time const. τ_p (ps)
NPT_short	2	Berendsen[S2]	0.1	Berendsen	0.1
NPT_long	10	Nose-Hoover[S3-S4]	1.0	Parrinello-Rahman[S5]	4.0
NVT_short	1	Berendsen	0.1		
NVT_long	5	Berendsen	0.5		

All results were derived from *NVT_long* simulations. In each case the TSSF-s were calculated also from the simulated models, using simulated partial radial distribution functions, by an in-house code. For calculating partial radial distribution functions the *g_rdf* software was used, that is part of the GROMACS software package.

Goodness-of-fit:

$R_W[F(Q)] = \sqrt{\frac{\sum_i (F^S(Q_i) - F^E(Q_i))^2}{\sum_i (F^E(Q_i))^2}}$, where $F^S(Q)$ is the calculated value from the MD simulation, $F^E(Q)$ is the experimental structure factor.

(S2) Berendsen, H. J. C.; Postma, J. P. M.; DiNola, A.; Haak, J. R. Molecular dynamics with coupling to an external bath. *J. Chem. Phys.* **1984,** 81, 3684.
(S3) Nose, S. A molecular dynamics method for simulations in the canonical ensemble. *Mol. Phys.* **1984,** 52, 255-268.
(S4) Hoover, W. G. Canonical dynamics: Equilibrium phase-space distributions. *Phys. Rev. A* **1985,** 31, 1695.
(S5) Parrinello, M.; Rahman, A. Polymorphic transitions in single crystals: A new molecular dynamics method. *J. Appl. Phys.* **1981,** 52, 7182.

Figure S2: Selected partial radial distribution functions for the mixture with 40 mol % ethanol as a function of temperature.

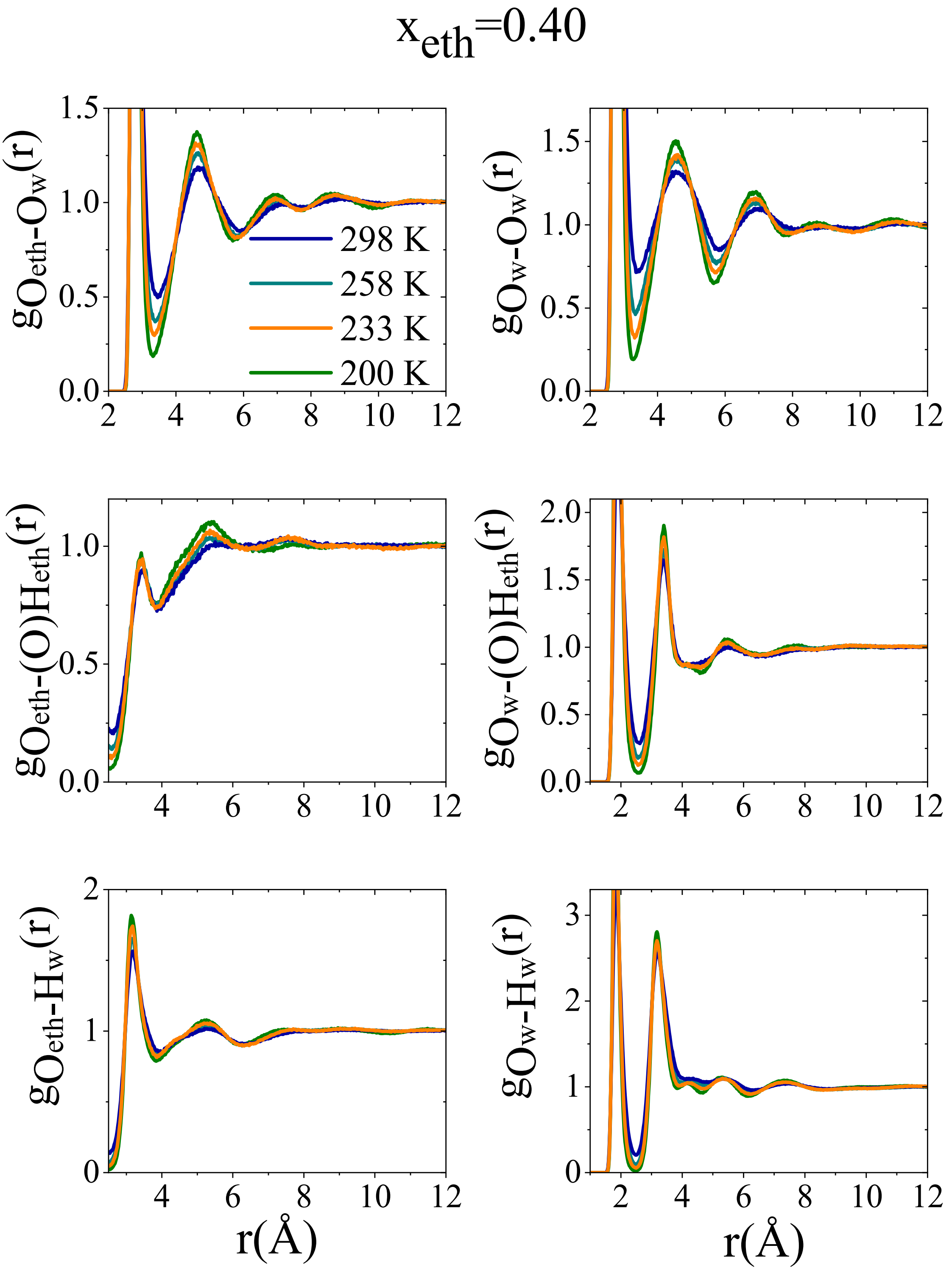

Figure S3: Selected partial radial distribution functions for the mixture with 50 mol % ethanol as a function of temperature.

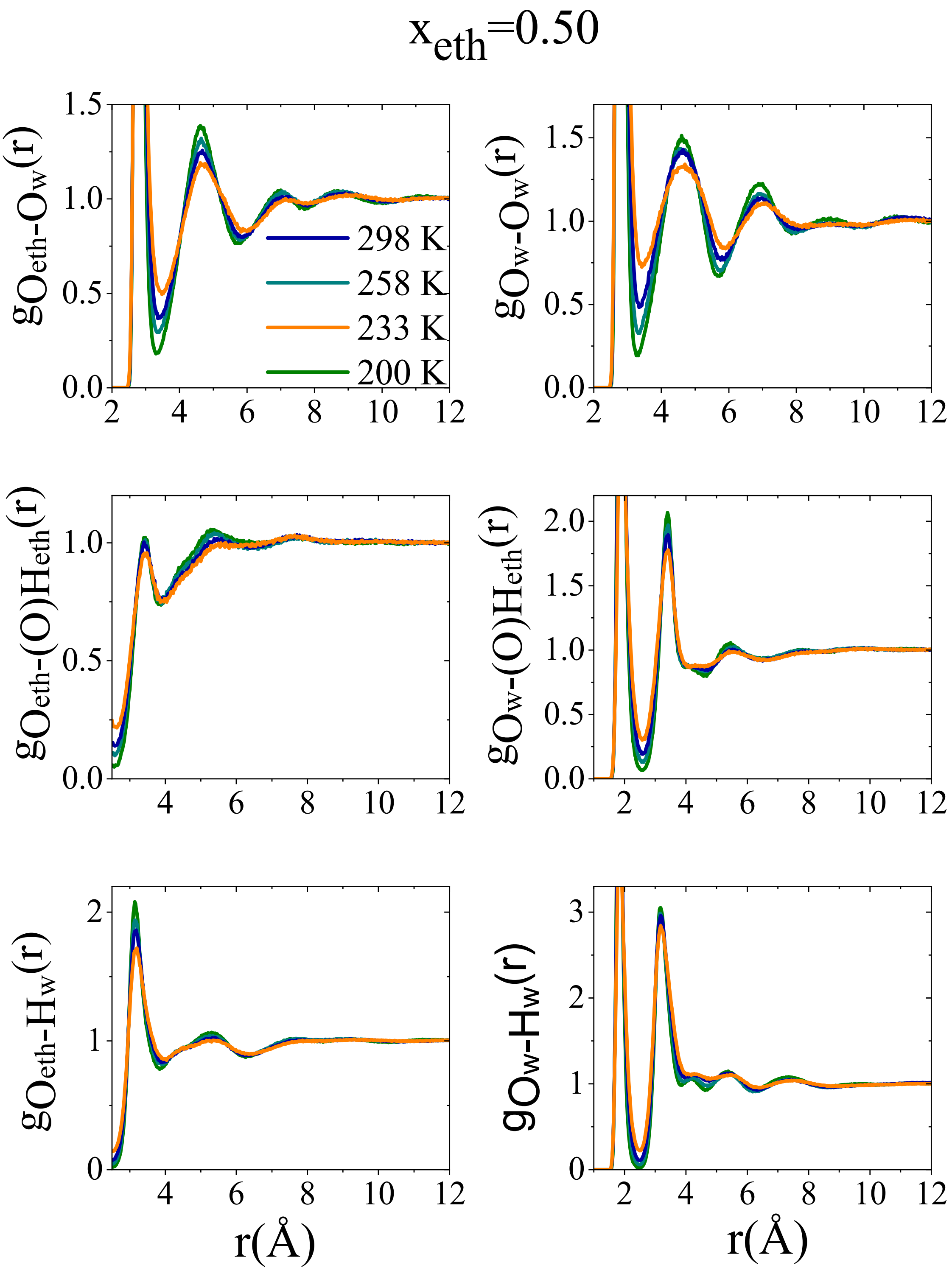

Figure S4: Selected partial radial distribution functions for the mixture with 60 mol % ethanol as a function of temperature.

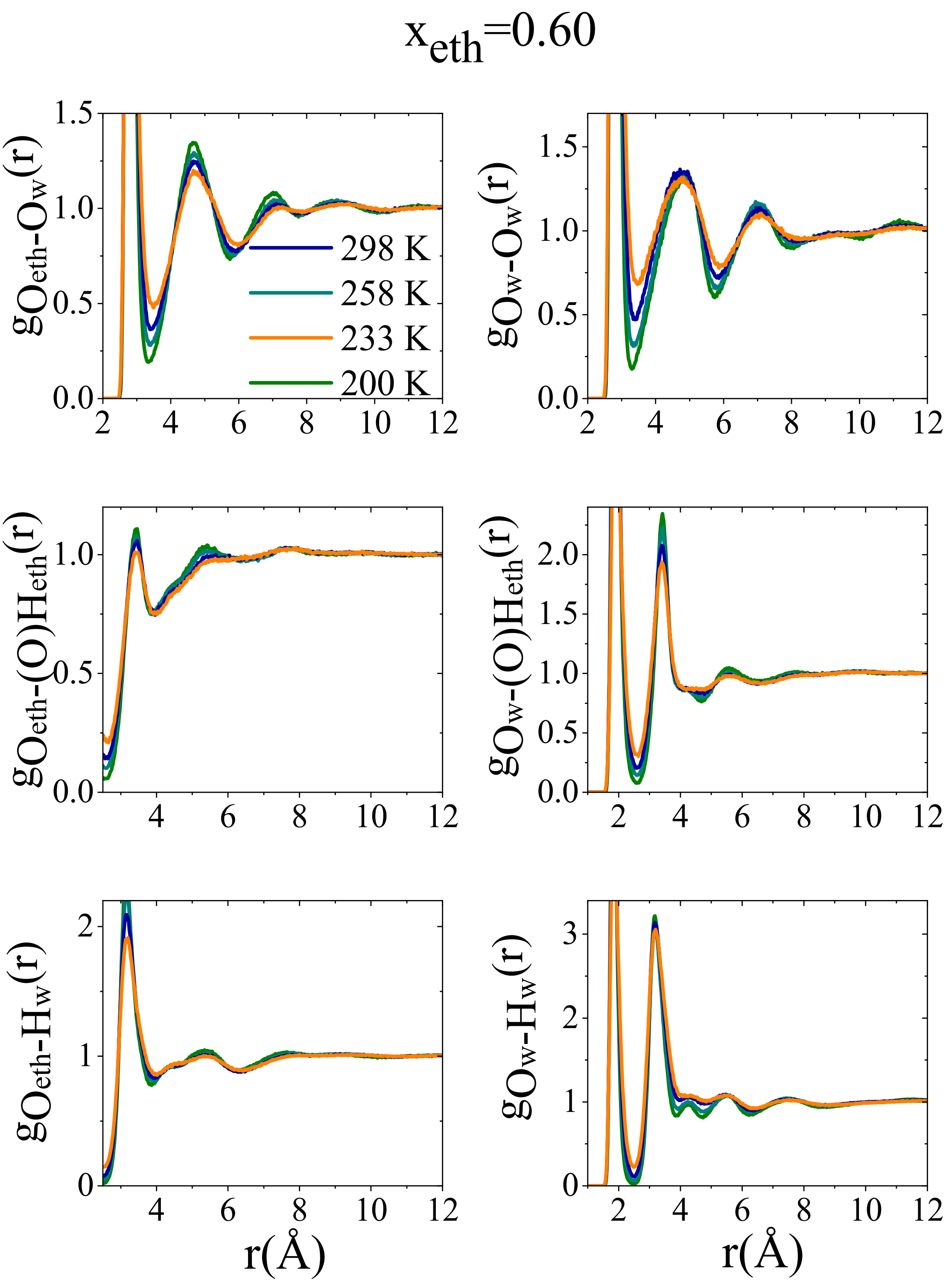

Figure S5: Selected partial radial distribution functions for the mixture with 70 mol % ethanol as a function of temperature.

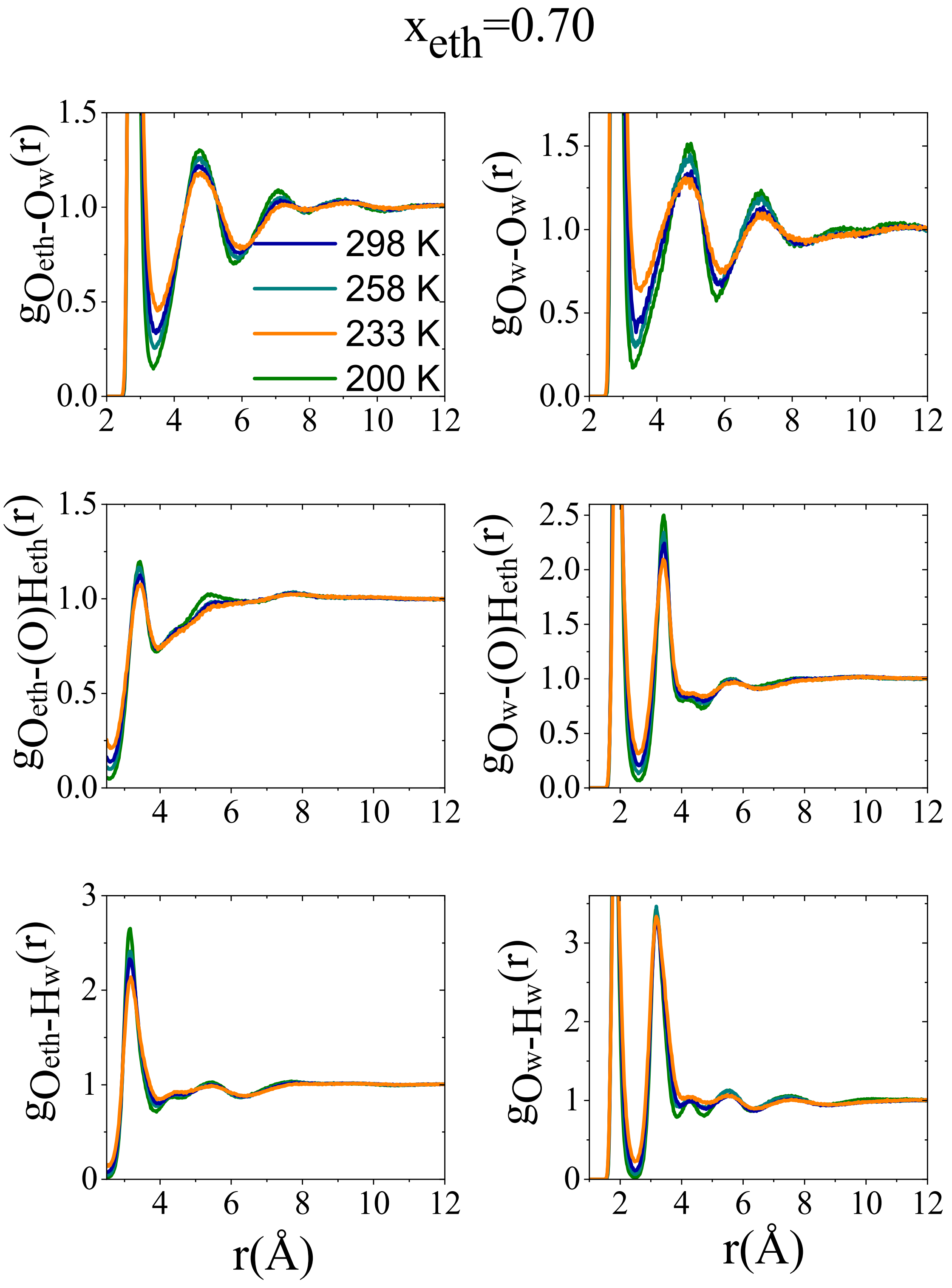

Figure S6: Selected partial radial distribution functions for the mixture with 80 mol % ethanol as a function of temperature.

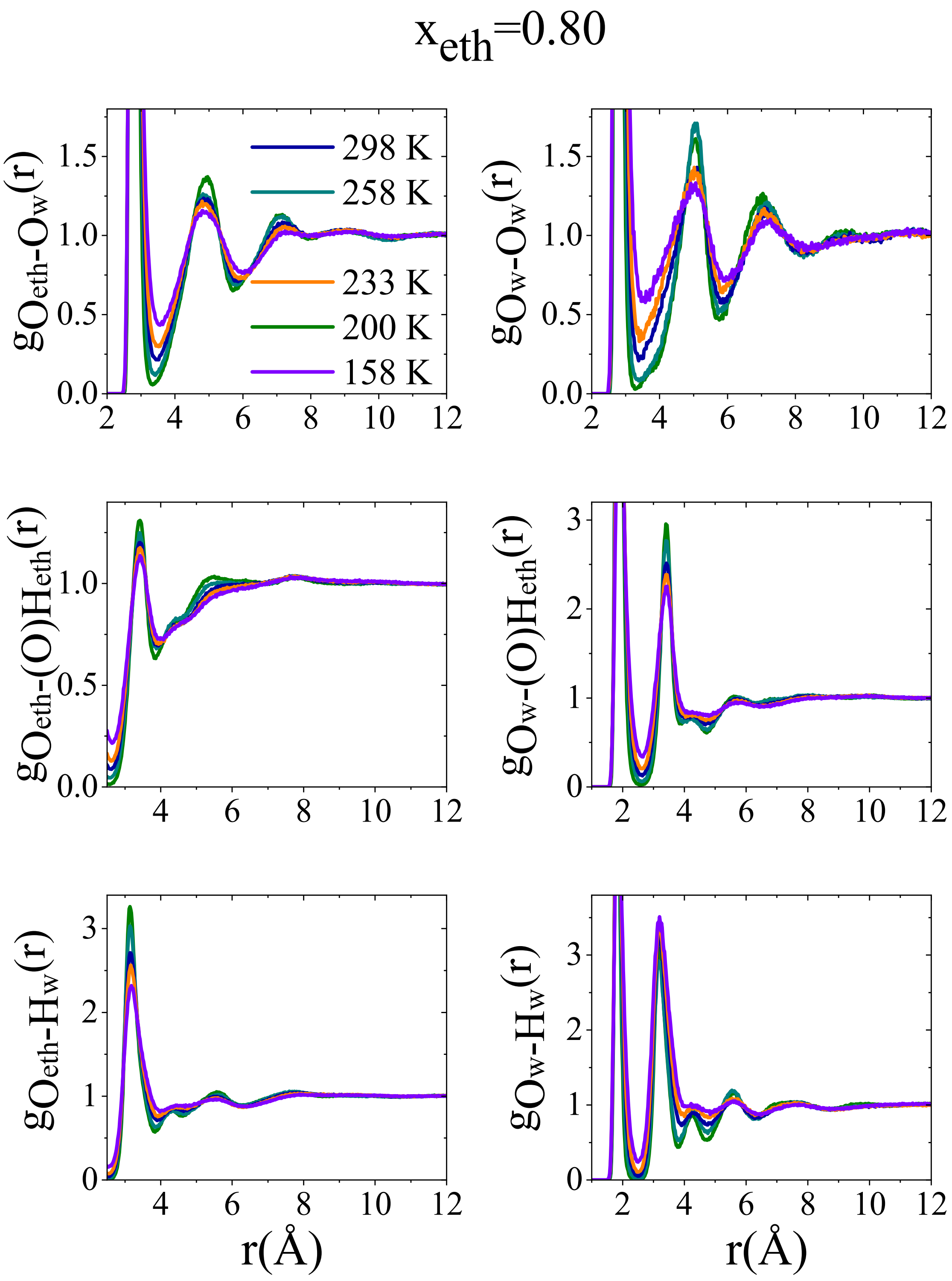

Figure S7: Selected partial radial distribution functions for the mixture with 85 mol % ethanol as a function of temperature.

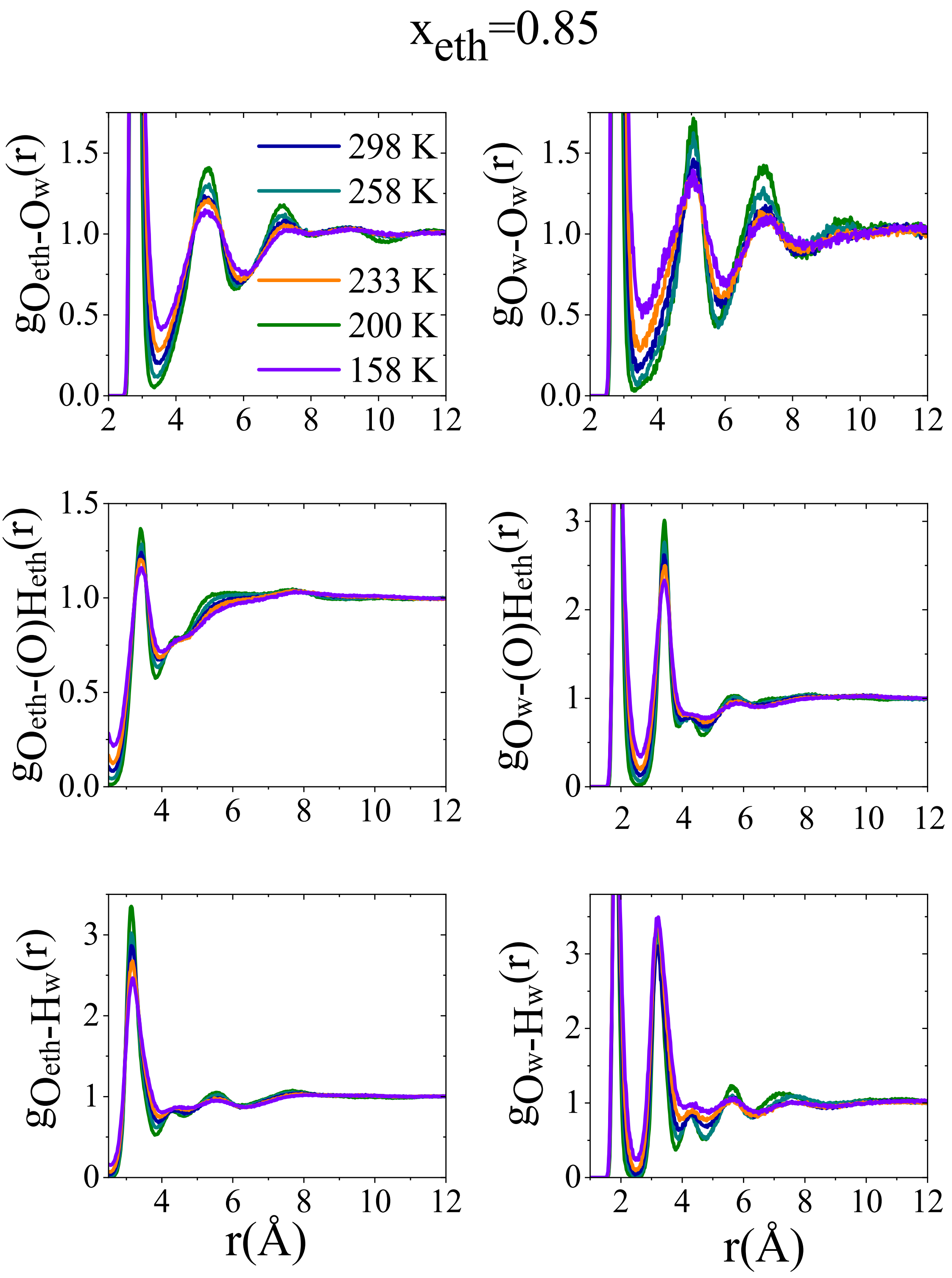

Figure S8: Selected partial radial distribution functions for the mixture with 90 mol % ethanol as a function of temperature.

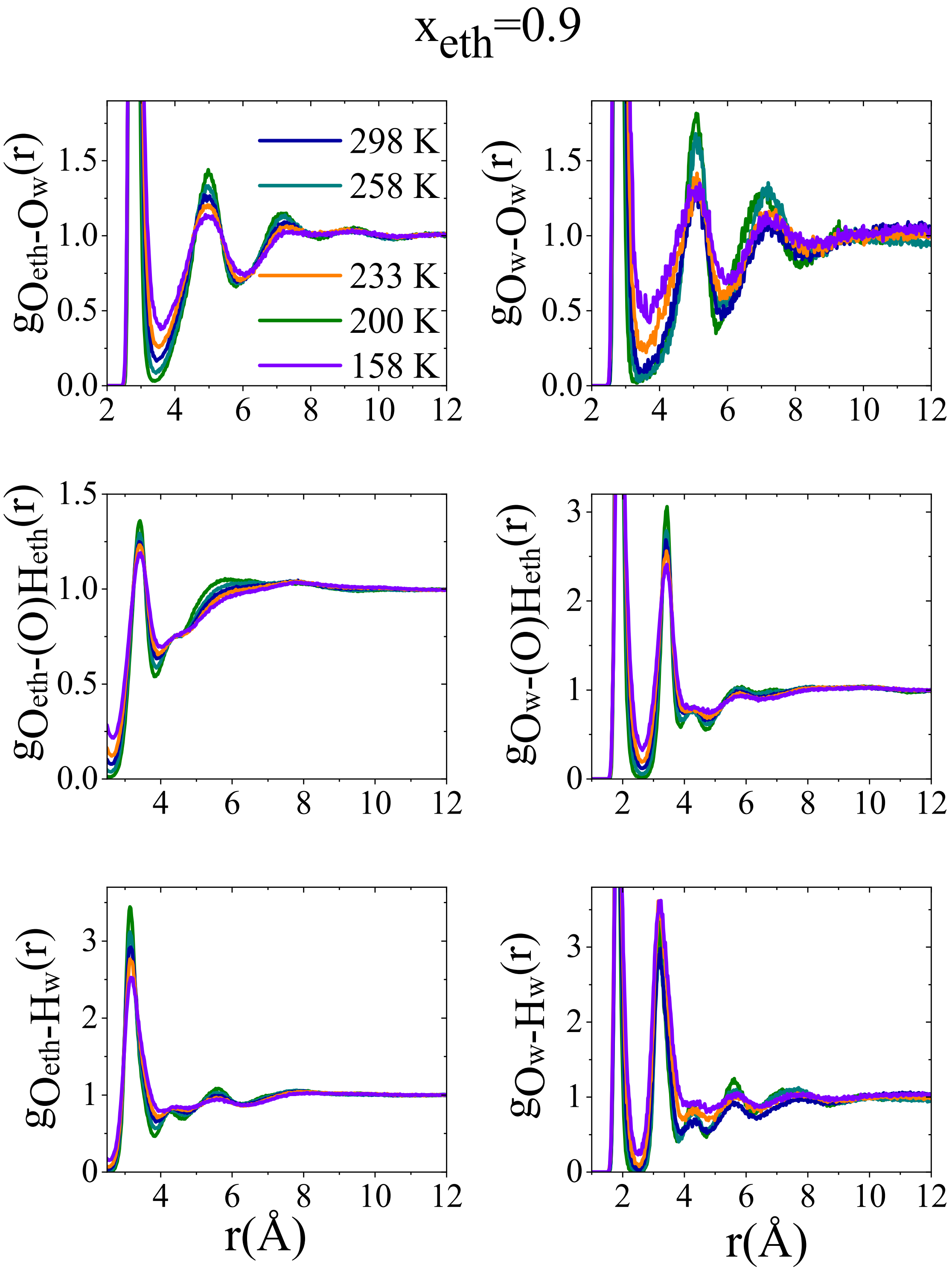

H-bond definition

Two molecules were considered hydrogen bonded (1) if they were found at a distance r(O···H) < 2.5 Å and H-O…O angle < 30° or (2) if they were found at a distance r(O···H) < 2.5 Å and the interaction energy between molecules was more negative than -3 kcal/mol (ca. -12 kJ/mol). In the latter case H-bond interactions are taken into account as attractive interactions. Note that results only with the "energetic" definition are presented in this work: the two definitions were in good agreement and the energetic definition, whenever it is available, is thought to be more robust.

The average number of hydrogen bonds (n_{HB}) in the mixtures (Figure S9), when taking into account all the connections, decreases when the ethanol content increases. At each concentration n_{HB} linearly increases with decreasing temperature. Water subsystems follow this tendency, but only at ethanol concentrations lower than 60 mol%. At higher concentrations the number of H-bonds between water pairs is almost constant. This latter statement is, independently from the ethanol concentration, also true for the ethanol subsystem (Fig. S10) over the entire temperature range investigated.

Figure S9. Average H-bond numbers considering each molecule, regardless of their types, together with the case when considering water–water H-bonds only.

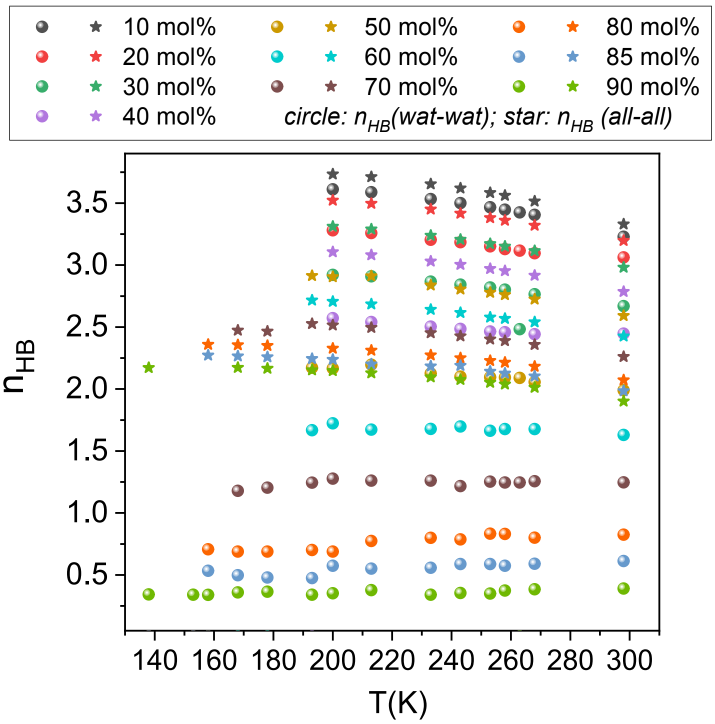

Figure S10: Average H-bond number for ethanol-ethanol subsystem.

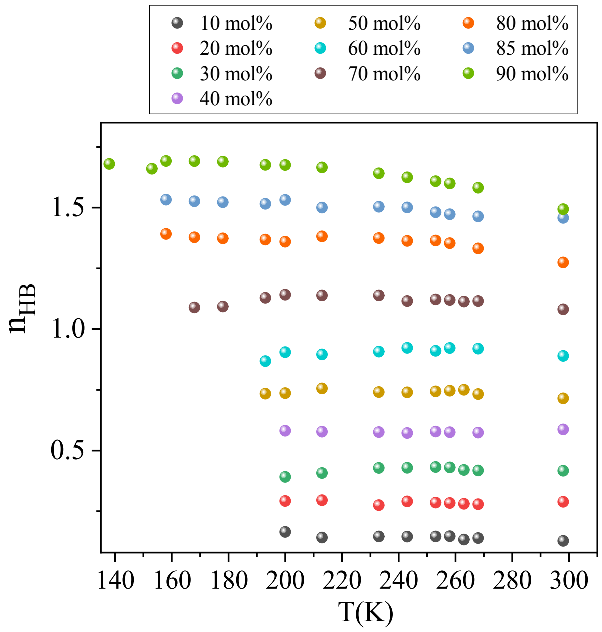

The number of H-bonded neighbors around (central) water and (central) ethanol molecules (Fig. S11) varies linearly with temperature at all concentrations. The only exception is the two highest ethanol concentrations (85 mol% and 90 mol%) below 190 K, where n_{HB} becomes constant.

Figure S11 Average H-bond numbers considering connections of water molecules only, as well as considering connections of ethanol molecules only.

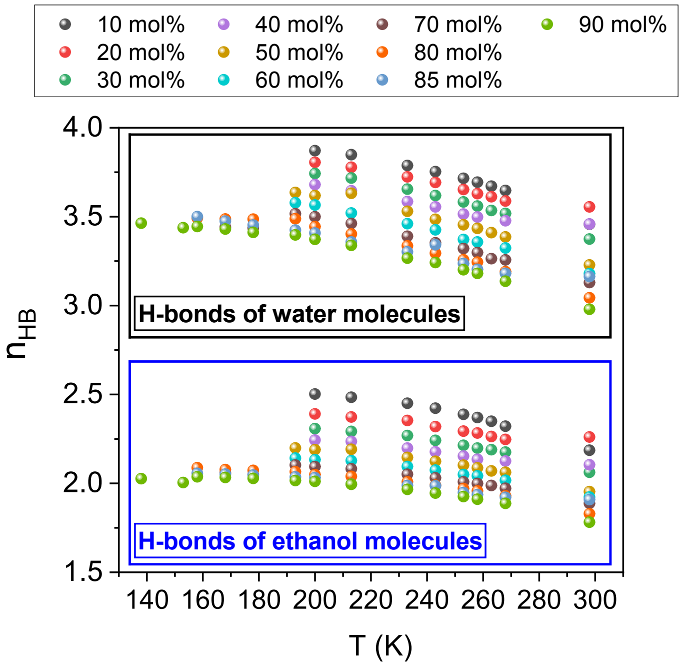

Figure S12: Fraction of donor and acceptor sites as a function of temperature: a) ‘1A:1D’ for ethanol molecules; b) ‘1D:2A’ for ethanol molecules; c) ‘2D:1A’ for water molecules; d) ‘2D:2A’ for water molecules.

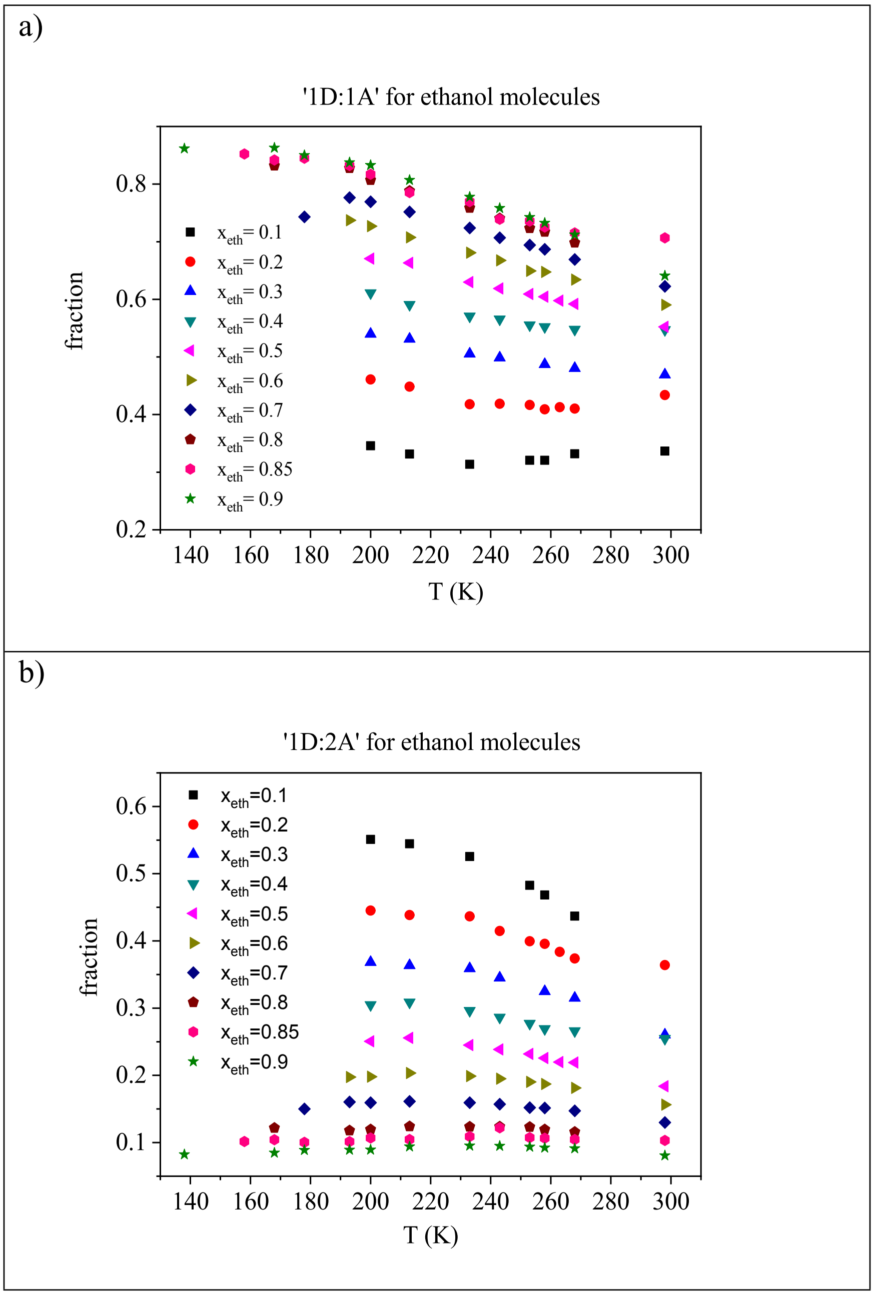

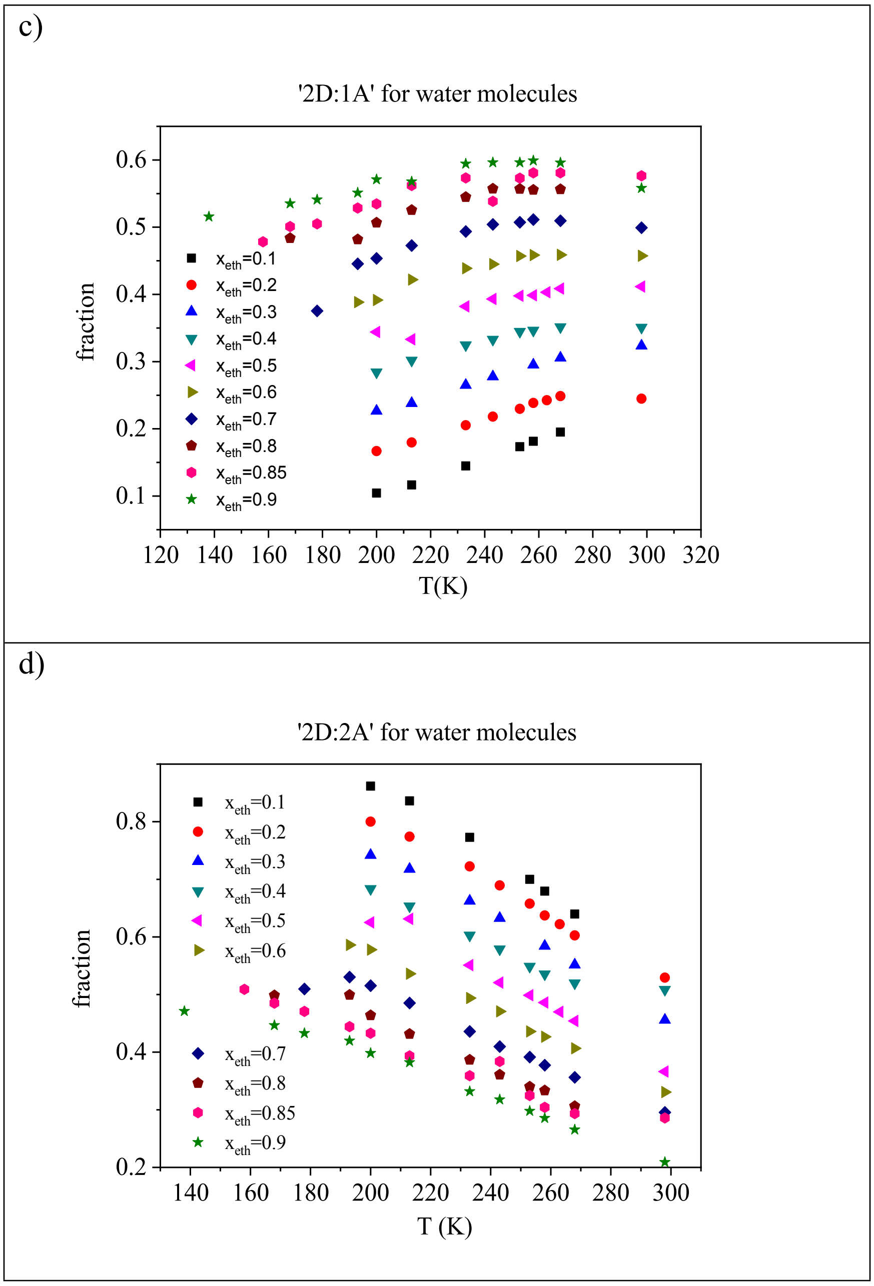

The behaviour of the ‘1A:1D’ and ‘1D:2A’ combinations for ethanol molecules, as well as that of the ‘2D:1A’ and 2D:2A’ combinations for water molecules, as a function of temperature, can be found in Fig. S12.

Concerning the ‘2D:2A’ combination as temperature decreases the occurrence of this scheme case linearly increases for water molecules, at every concentration: this may be taken as an indication that ‘2D:2A’ represents an inherent arrangement, being the most stable according to potential energy considerations.

H-bond number excess parameter

There are several approaches[S6-S8] for capturing deviations from characteristics of a system with randomly distributed molecules. The basic question is whether some type(s) of hydrogen bonds (e.g. water-water, water-ethanol, etc…) are preferred (i.e. more frequent than they are in a randomly distributed system). The following parameter is defined for characterizing a (possibly preferential) H-bonded environment around a central molecule:

$$f_{\alpha\beta} = \frac{n_{\alpha\beta}}{n_{\alpha all}} \frac{1}{x_\beta} \quad , \tag{1}$$

where $n_{\alpha\beta}$ and $n_{\alpha all}$ is the average H-bond number between α – β and α - all (α+β) pairs, respectively. x_β is the mole fraction of component β. In the case of an ideal (totally random) ethanol-water mixture this $f_{\alpha\beta}$ value is 1.0, whereas values higher than unity indicate preferential H-bonding.

Results are presented in Fig. S13. The $f_{wat\text{-}eth}$ and $f_{eth\text{-}eth}$ functions increase almost linearly with the increasing ethanol content. For $f_{eth\text{-}eth}$ a noticeable minimum can be detected at x_{eth}=0.3. On the other hand, in the case of $f_{wat\text{-}eth}$ a maximum emerges at x_{eth}=0.6. The $f_{wat\text{-}wat}$ function is above 1.0 over almost the entire concentration range. A well-defined maximum can be identified for $f_{wat\text{-}wat}$ around ethanol mole fractions 0.5-0.6 at 298 K, which is shifted at 200 K to ethanol mole fractions of 0.6-0.7. This corresponds to a significant excess of water molecules in the solvation shell of water. This maximum agrees well with the maximum of $G_{wat\text{-}wat}$ in Kirkwood-Buff integral theory.[S9-S11]

(S6) Miroshnichenko, S.; Vrabec, J. Excess properties of non-ideal binary mixtures containing water, methanol and ethanol by molecular simulation. *J. Mol. Liq.* **2015**, 212, 90-95.
(S7) Soetens, J-C.; Bopp P. A. Water−Methanol Mixtures: Simulations of Mixing Properties over the Entire Range of Mole Fractions. *J. Phys. Chem. B* **2015**, 119, 8593−8599.
(S8) Vlček, L.; Nezbeda, I. Excess Properties of Aqueous Mixtures of Methanol: Simple Models versus Experiment. *J. Mol. Liq.* **2007**, 131, 158−162.
(S9) E. Matteoli; L. Lepori. Solute–solute interactions in water. II. An analysis through the Kirkwood–Buff integrals for 14 organic solutes. *J. Chem. Phys.* **1984**, 80, 2856.
(S10) Perera, A.; Sokolić, F.; Almásy, L.; Koga, Y. Kirkwood-Buff integrals of aqueous alcohol binary mixtures. *J. Chem. Phys.* **2006**, 124, 124515.
(S11) Ben-Naim, A. Inversion of the Kirkwood–Buff theory of solutions: application to the water–ethanol system. *J. Chem. Phys.* **1977**, 67, 4884.

Figure S13. H-bond number excess parameter: parameter for characterizing the preferential H-bonded environment around a central molecule ($f_{\alpha\beta}$). Black solid circle symbols: $f_{wat\text{-}wat}$ at 298 K; red open circle symbols: $f_{wat\text{-}wat}$ at 200 K; black solid triangle symbols: $f_{wat\text{-}eth}$ at 298 K; green open triangle symbols: $f_{wat\text{-}eth}$ at 200 K; black solid square symbols: $f_{eth\text{-}eth}$ at 298 K; blue open square symbols: $f_{eth\text{-}eth}$ at 200 K.

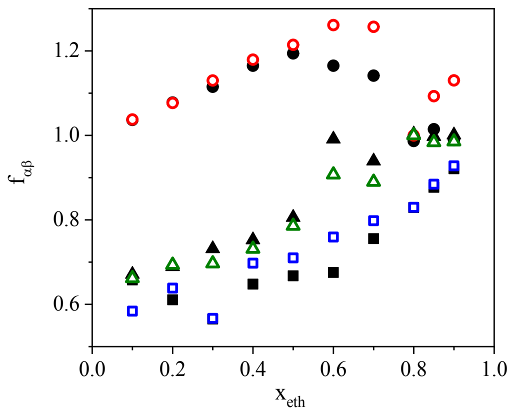

Figure S14. The average largest cluster size (C1) and average second largest cluster size (C2) as a function of ethanol concentration and temperatures in ethanol-water mixtures. Violet sphere symbols: x_{eth}=0.1; orange sphere symbols: x_{eth}=0.2; navy sphere symbols: x_{eth}=0.3; dark cyan sphere symbols: x_{eth}=0.4; magenta sphere symbols: x_{eth}=0.5; black sphere symbols: x_{eth}=0.6; green sphere symbols: x_{eth}=0.7; red sphere symbols: x_{eth}=0.8; blue sphere symbols: x_{eth}=0.85; dark red sphere symbols: x_{eth}=0.9.

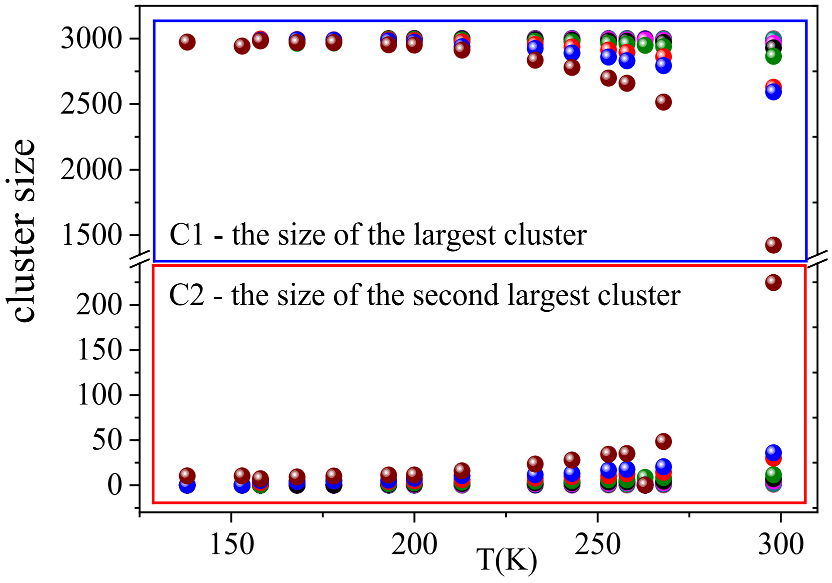

The average largest cluster size (C1) and average second largest cluster size (C2) are plotted in Fig. S14. Several works[S12-S15] have already proved that the properties of these quantities are good indicators for determining the location (concentration-temperature pair) of the percolation transition. At low temperatures, below 230 K, the number of molecules in the largest cluster equals the total number of molecules in the systems. It also means that the value of C2 is almost zero at each studied concentration. In these cases, molecules percolate throughout the systems. Around 230 K the spheres corresponding to the different concentrations start to move away from each other, i.e., the largest clusters start to shrink. The largest cluster at x_{eth}=0.9 and 300 K is only half the size of what it was at 150 K, which means that percolation is questionable. The average second largest cluster size shows that the system contains several smaller assemblies of less than 200 molecules. Note that the only case where the question of existence of percolation threshold arises is the 90 mol% solution at room temperature.

Fractal dimension of the largest cluster:

According to random site percolation theory, infinite clusters are true fractals at the percolation threshold with fractal dimension f_d=2.53 in three dimensions, and f_d=1.896 in two dimensions.[S12-S14] It has already been shown[S12-S14] that we cannot detect a percolated cluster with f_d value smaller than 2.53 in three, and 1.896 in two dimensions.

In the light of the foregoing, among the systems studied the one with 90 mol% ethanol deserves further scrutinization from the point of view of the percolation threshold. It was found that the *fractal dimension of the largest cluster* is 2.73 at 298 and 2.90 at 200 K, respectively. These values are significantly larger than the corresponding values to the percolation threshold. It can be stated that the largest cluster forms a 3D percolated network still at x_{eth}=0.9. For the other systems studied, f_d values were larger than 2.8, which confirms that their largest clusters form 3D percolated networks.

(S12) Pártay, L. B.; Jedlovszky, P.; Brovchenko, I.; Oleinikova, A. Percolation Transition in Supercritical Water: A Monte Carlo Simulation Study. *J. Phys. Chem. B* 2007, 111 7603-7609.

(S13) Pártay, L. B.; Jedlovszky, P.; Brovchenko I.; Oleinikova, A. Formation of mesoscopic water networks in aqueous systems. *Phys. Chem. Chem. Phys.* **2007**, 9, 1341–1346.

(S14) Xu, X.; Wang, J.; Lv, J.-P.; Deng, Y. Simultaneous analysis of three-dimensional percolation models. *Front. Phys.* **2014**, 9(1), 113–119.

(S15) Bakó, I.; Oláh, J.; Lábas, A.; Bálint, Sz.; Pusztai, L.; Bellissent-Funel, M.-C. Water-formamide mixtures: Topology of the hydrogen-bonded network. *J. Mol. Liq.* **2017**, 228, 25-31.

Figure S15. Cluster size distributions from the room temperature to the lowest studied temperature a) for x_{eth}=0.85, b) for pure ethanol.

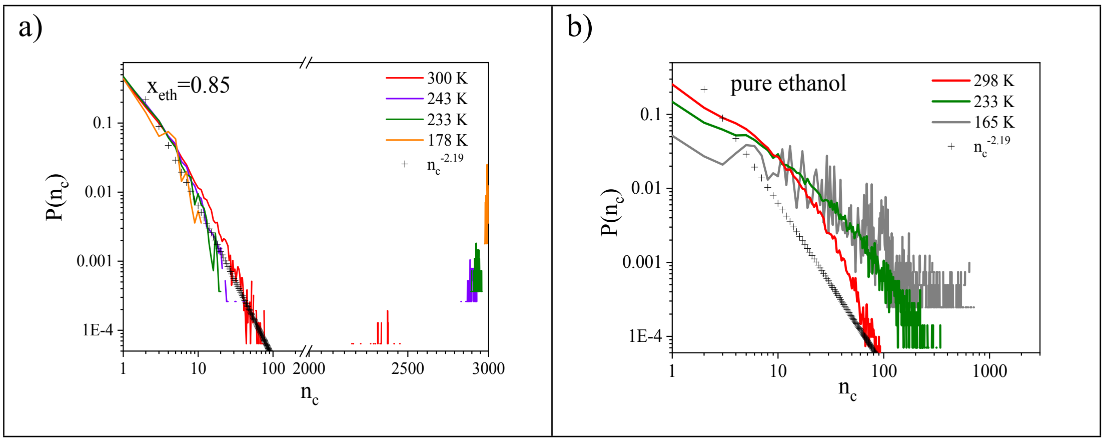

Figure S16. Typical hydrogen bonded network topologies in water-ethanol mixtures at concentrations x_{eth} = 0.40 (left), 0.70 (middle), and 0.90 (right). Red symbols indicate ethanol molecules, green symbols represent water molecules, while lines between molecules are the H-bonds.

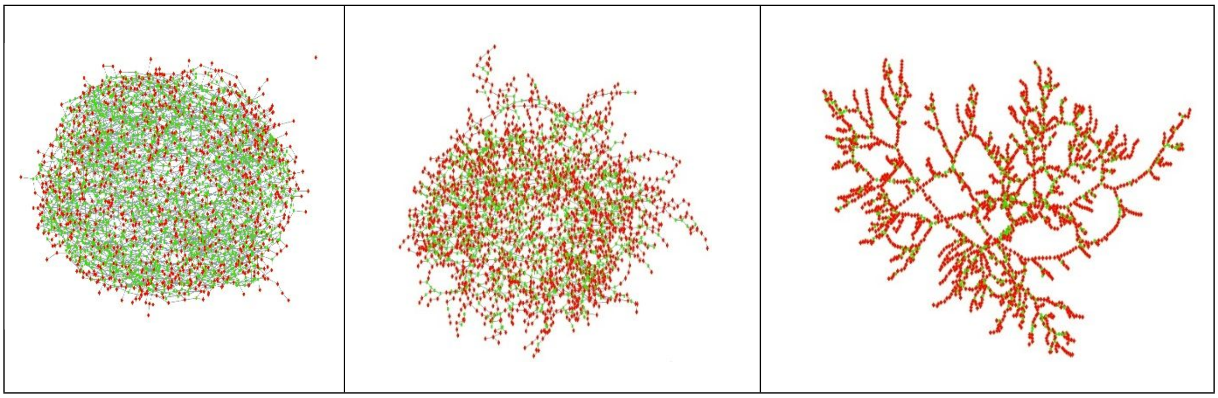

At low ethanol concentrations, these systems contain mainly cyclic entities, whereas at higher alcohol concentrations more and more chains appear. For x_{eth}=0.9, even a dendrite type structure can be found that exists, for instance, in the neural networks.[S16,S17]

The above pictures represent single H-bonded clusters, shown here as examples. Apart from the mixtures with high alcohol content, below the percolation limit, the clusters represent practically the entire system. Identification of small (5- or 6-membered) rings is not done from these pictures, but by suitable algorithms coded into our software[S18].

(S16) Rall, W. Branching dendritic trees and motoneuron membrane resistivity. *Exp Neurol.* **1959**, 1, 491-527.
(S17) Koch, C.; Poggio, T.; Torre, V. Nonlinear interactions in a dendritic tree: localization, timing, and role in information processing. Proc. Natl. Acad. Sci. USA. 1983, 80(9), 2799-802.
(S18) Bakó, I.; Megyes, T.; Bálint, Sz.; Grósz, T.; Chihaia, V. Water–methanol mixtures: topology of hydrogen bonded network. *Phys. Chem. Chem. Phys.* **2008,** 10, 5004-5011.

Background of the Laplace spectra

The structure of a network can be fully characterized with the adjacency or the combinatorial Laplace (L) matrices.[S19-S30] The Laplace matrix can be defined as follows:

$$L_{ij} = k_i \delta_{ij} - A_{ij} \tag{1}$$

where k_i is the number of (hydrogen) bonded neighbours of molecule 'i'; δ_{ij} is the Kronecker delta function and A_{ij}=1 if a bond exists between nodes i and j.

It is known that the Laplacian matrix is positive semidefinite and has nonnegative eigenvalues.[S19,S20] Furthermore, 0 is always an eigenvalue of L and the multiplicity of the eigenvalue 0 is equal to the number of the connected components of the graph.

(S19) Van Mieghem, P. Graph Spectra for Complex Networks. Cambridge University Press, 2010.
(S20) Cvetkovic, D.; Simic, S. Graph spectra in Computer Science. *Linear Algebra Appl.* **2011**, 434, 1545–1562.
(S21) Von Luxburg, U. A tutorial on spectral clustering. *Stat. Comput.* **2007**, 17, 395-416.
(S22) Chung, F. Spectral Graph Theory. Chapter 2.2. A.M.S. CBMS, Providence, Rhode Island, 1997.
(S23) Banerjee, A.; Jost, J. On the spectrum of the normalized graph Laplacian. *Linear Algebra Appl.* **2008**, 428, 3015-3022.
(S24) McGraw, P. N.; Menzinger, M. Laplacian spectra as a diagnostic tool for network structure and dynamics. *Phys. Rev. E* **2008**, 77, 031102.
(S25) Hata, S.; Nakao, H. Localization of Laplacian eigenvectors on random networks. *Sci. Rep.* **2017,** 7, 1121.
(S26) Julaiti, A.; Wu, B.; Zhang, Z. Eigenvalues of normalized Laplacian matrices of fractal trees and dendrimers: analytical results and applications. *J. Chem. Phys*. **2013**, 138, 204116.
(S27) de Abreu, N. M. M. Old and new results on algebraic connectivity of graphs. *Linear Algebra Appl.* **2007**, 423, 53–73.
(S28) Fiedler, M. A property of eigenvectors of nonnegative symmetric matrices and its applications to graph theory. *Czech. Math. J.* **1975**, 25, 619-633.
(S29) Friedman, J. Some geometric aspects of graphs and their eigenfunctions. *Duke Math J.* **1993**, 69, 487-525.
(S30) Bakó, I.; Pethes, I.; Pothoczki, Sz.; Pusztai, L. Temperature dependent network stability in simple alcohols and pure water: The evolution of Laplace spectra. *J. Mol. Liq.* **2019**, 273, 670–675.

‘Stability’ of H-bonded networks

Finally, we would like to provide some indicator for the ‘stability’ of H-bonded networks found in ethanol-water mixtures, as a function of temperature. Below we wish to devise a simple number that is related of the number of H-bonds that needed to be removed so that the network in question would not be percolating any longer.

The most important theorem is coupling to the connectivity and to the second smallest positive eigenvalue (Fiedler eigenvalue) of a Laplacian is known as the Cheeger inequality [S31, S32]:

$$\frac{\lambda_2}{2} < h(G) < \sqrt{2\lambda_2} \quad (2)$$

where h(G) is the Cheeger constant (or conductance) of a graph G. This inequality is related to the minimum number of bonds such that, when removed, cause the graph to become disconnected (‘non-percolated’ according to the terminology of H-bonded networks) [S32]. Therefore, h(G) (or a similarly derived quantity like $\lambda_2 * n_{HB,}$ see below) can serve as a well-defined parameter to measure the ‘distance’ from the percolation transition. Some applications of this theory for molecular liquid can be found in Ref. S18. and S34-S36.

The h(G) quantity is defined by the following equation.

$$h(G) = \min \frac{E(Sc, V - Sc)}{\min(Vol(Sc), Vol(V - Sc))} \quad (3)$$

Here, $V–S_C$ and S_C are two non-empty subsets of V nodes of the G graph, $Vol(S_C)$ and $Vol(V–S_C)$ are the sums of the number of both intra- and inter-set connections of each node of the given subset. The $E(V–S_{C,}\ S_C)$ is the number of inter-set links, connect nodes belonging to the

(S31) Fiedler, M. A property of eigenvectors of nonnegative symmetric matrices and its applications to graph theory. *Czech. Math. J.* **1975**, 25, 619-633.
(S32) Friedman, J. Some geometric aspects of graphs and their eigenfunctions. *Duke Math J.* **1993**, 69, 487-525.
(S33) Choi, S.; Parameswaran, S; Choi, J-H. Understanding alcohol aggregates and the water hydrogen bond network towards miscibility in alcohol solutions: graph theoretical analysis. *Phys. Chem. Chem. Phys* **2020**, **22**, 17181-17195.
(S34) Bakó, I.; Pethes, I.; Pothoczki, Sz.; Pusztai, L. Temperature dependent network stability in simple alcohols and pure water: The evolution of Laplace spectra. *J. Mol. Liq.* **2019**, 273, 670–675.
(S35) Choi, J.-H.; Choi, H. R.; Jeon, J.; Cho, M. Ion aggregation in high salt solutions. VII. The effect of cations on the structures of ion aggregates and water hydrogen-bonding network. *J. Chem. Phys.* **2017**,147, 154107.
(S36) Choi, J.-H.; Cho, M. Ion aggregation in high salt solutions. V. Graph entropy analyses of ion aggregate structure and water hydrogen bonding network. *J. Chem. Phys*. **2016**, 144, 204126.

different subsets. This expression has a minimum if the number of inter-set links (i.e. bonds) is taken as 1 and the denominator is a value indicating the half of the "volume" size of the entire system (Vol(V)/2, equals to the number of links of the G graph or by other words: the total number of hydrogen bonds in the configuration). We can call this value $h(G)_{min}$. After simple mathematical transformations, we can obtain the following inequality from Eq. 2.:

$$\frac{\lambda 2}{2h(G)min} \leq \frac{h(G)}{h(G)min} \leq \frac{\sqrt{2\lambda 2}}{h(G)min} \quad (4)$$

The left and the right sides of the inequality defined by Equation 4 are shown in Figure S17. This inequality is related to the minimum number of bonds that, when removed, cause the graph to become disconnected ('non-percolated'). This inequality provides a lower and upper limit on the stability of the percolated network, considering also the effect of finite size. It can be seen that the stability of the hydrogen bond network decreases significantly with increasing ethanol concentration.

Figure S17. Values of the inequality calculated by Equation 4. Black open squares: left side of Eq. 4 at 298 K; black solid squares: right side of Eq. 4 at 298 K; red open circles: left side of Eq. 4 at 233 K; red solid circles: right side of Eq. 4. at 233 K.

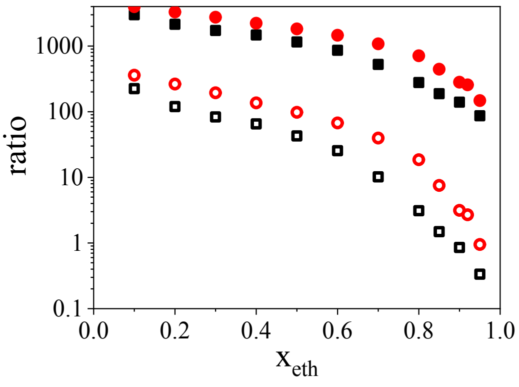

Figure S18. Composition dependence of the smallest λ_2 value. Red line: an estimate of h(G) ($2/N^*n_{HB}$). The blue arrow points to where percolation transition occurs.

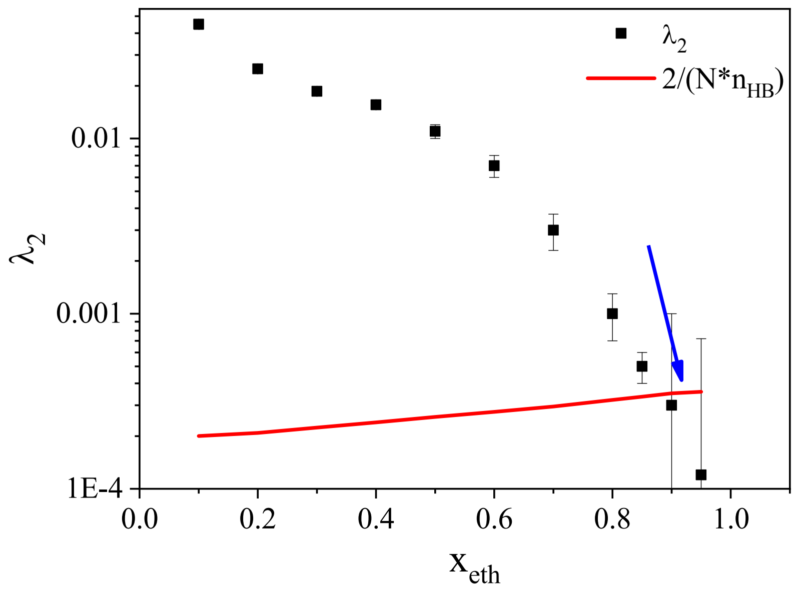

As another demonstration of the power of Laplace-spectra, in Figure S18 the composition dependence of (the smallest) λ_2 is shown, together with a simple estimation of h(G). According to the (consequences of the) Cheeger equation (see above), wherever h(G) is larger than the smallest λ_2, taking away just one single hydrogen bond causes the percolating network to destabilize. According to Figure S18, the percolation threshold is around x_{eth}=0.9 (see the blue arrow in Figure S18).
